# Students' relationship quality in class: Exploring latent profiles, latent transitions and links to student motivation

**DOI:** 10.1111/bjep.70028

**Published:** 2025-09-10

**Authors:** Juliane Schlesier, Ramona Obermeier, Michaela Gläser‐Zikuda

**Affiliations:** ^1^ Empirical Research on Learning and Instruction CvO University of Oldenburg Oldenburg Germany; ^2^ Department for Educational Research Linz School of Education, JKU Linz Linz Austria; ^3^ Department of Educational Science, School Education and Instructional Research Friedrich‐Alexander‐University of Erlangen‐Nürnberg Nürnberg Germany

**Keywords:** latent profile analysis, latent transition analysis, motivation, peer relationships, teacher–student relationships

## Abstract

**Background:**

Early secondary school students' perceptions of their social relationships in class are closely linked to their levels of motivation. However, person‐centred research examining patterns of social relationships with both peers and teachers remains limited.

**Aims:**

This study explored the existence, stability and transitions of latent profiles of students' social relationships over time, as well as their interconnectedness with students' motivation, gender, immigration background and educational track.

**Sample(s):**

The sample included 1343 5th grade students (first year after transitioning to secondary school), assessed at three time measurement points.

**Methods:**

Latent profile analysis (LPA) was used to identify patterns of students' perceived social relationship quality. We also used latent transition analysis (LTA) to explore the stability and transitions between profiles over time, taking into account students' motivation, gender, immigration background and educational track.

**Results:**

Four profiles of social relationship quality were identified at each time point: low (P1), moderate (P2), high (P3) and solid (P4). Profiles with lower‐quality relationships were the least stable across time. Intrinsic motivation was a key predictor of profile transitions, with higher motivation increasing the likelihood of moving to profiles with lower‐quality relationships. Gender, immigration background and educational track influenced initial profile assignment significantly.

**Conclusions:**

The findings highlight the dynamic nature of perceived social relationships in early secondary school. Interventions that enhance intrinsic motivation might be useful in promoting students' social development.

## INTRODUCTION

Social relatedness and the quality of social relationships are not only crucial for the emotional experiences of children and adolescents (Becker & Luthar, 2002; Kelly & Malecki, 2022; Lei et al., 2018; Mainhard et al., 2018; Mai et al., 2021; Markus et al., 2022), but also for their behavioural and performance outcomes at school (Morinaj & Hascher, 2022; Wentzel, 2017; Yeung & Leadbeater, 2010). For this reason, students' social relationships and the importance of these for their lives and outcomes have been the subject of numerous research studies in recent years. Research studies have already identified connections between students' social relationships and their motivation for learning, suggesting that positive social relationships are essential for students being intrinsically motivated to learn and to engage at school (Burns et al., 2022; Harter, 1996; Lazarides et al., 2022; Raufelder et al., 2013; Roorda et al., 2011; Song et al., 2015). However, research on students' social relationships in the school environment is – until now – based primarily on variable‐centred approaches and rarely takes into account individual patterns of social relationships in school (Cornelius‐White, 2007; Lietaert et al., 2015; Roorda et al., 2011). The rare research studies that have examined social relationships in the school environment in connection with student motivation while also adopting a person‐centred approach refer either to motivational profiles (Gillet et al., 2017; Lazarides et al., 2021; Raufelder et al., 2022); or they mix social relationships with motivation in the profiles (Raufelder et al., 2013); or they do not refer to the important phase of the transition to secondary school (Maulana et al., 2011).

It is notable that the transition to secondary school – and particularly the post‐transition adjustment phase in the first year of secondary school – is a critical developmental phase during which students face significant changes in social relationships, including forming new peer groups and adapting to multiple teachers (Hughes & Cao, 2018; Spilt et al., 2012). Studying latent social relationship profiles and transitions during this adjustment period could help to identify patterns that support or hinder developing positive relationships, thus offering valuable guidance for interventions to ease students' transitions and adjustment to the new school environment. Moreover, by focusing on distinct latent profiles, a more nuanced understanding of how combinations of different aspects of social relationships may be related to students' levels of intrinsic and extrinsic motivation can be provided. While such studies are less common, they are essential for addressing research questions that involve groups with diverse characteristics (e.g., gender, immigration background, educational track) and, as mentioned before, for designing targeted, effective interventions.

Without including other related variables, student relationship profiles make it possible to provide precise starting points for improving the quality of relationships in early secondary school – and thus student motivation. For this reason, the present study aimed to determine profiles of the quality of perceived relationships between students and their teachers and peers and also to examine whether the students in particular profiles differ in terms of their intrinsic and extrinsic motivation. In addition, we investigated whether, and to what extent, the students are stable in their relationship profiles throughout the 5th grade after transitioning from primary to secondary school, as this time period tends to be accompanied by a change in student motivation, as well as in their social school context (Raufelder et al., 2022; Schlesier, Raufelder, & Moschner, 2023).

### Students' social relationships in school

In the school environment, students' relationships with peers and teachers are particularly important (Schlesier & Raufelder, 2025). Both these types of relationships play a vital role in students' level of school engagement, behavioural outcomes and ongoing academic success (Hoferichter et al., 2021; Morinaj & Hascher, 2022; Roorda et al., 2011; Schmidt et al., 2021; Shin & Chang, 2022; Wentzel, 2017; Yeung & Leadbeater, 2010). At the same time, they are closely interwoven with each other, and the individual needs and emotions of the people involved, that is, students and teachers (Hughes & Im, 2016; Schlesier & Raufelder, 2025). In the following sub‐sections, both relationships are first introduced separately, followed by an overview of their interplay with student motivation.

#### The student–student relationship

Hardly any other relationship is as important for primary and secondary school students as their relationship with their peers in class (Alivernini et al., 2019). Student relationships with other students differ from those with their teachers (who are adults), as students are on an equal footing with each other. Peer relationships can be formed as a dyadic relation, a clique with a few other students or a (class) crowd relationship (Brown & Klute, 2003; Zander et al., 2017). These relationships can be different in their constitutional roots – there are *affective* relationships that accompany (positive or negative) emotions in students, and *cognitive instrumental* peer relationships that refer to learning‐oriented exchanges between students (Zander et al., 2017).

Positive affective peer relationships in class are driven by reciprocal friendships and sympathy, shared goals, shared support and emotional security (Bukowski & Raufelder, 2018). Such peer relationships are particularly relevant when it comes to experiencing positive achievement emotions such as joy and pride, as such emotions are shared with the peers so that the students feel encouraged and can act out their emotions externally (Schlesier et al., 2024). Negative affective peer relationships are characterized by negative emotions that may emerge from bullying or other types of conflicts among students (Markkanen et al., 2021; Olweus, 1997). These negatively connoted relationships are particularly relevant because they are associated with an increased level of anxiety among students and are one of the push factors that can even cause students to drop out of school (Gubbels et al., 2019; Schlesier, Vierbuchen, & Matzner, 2023; Stearns & Glennie, 2006). Transition phases are particularly relevant in this context, as students' social environments at school are usually almost completely (or at least in part) reformed after transition; this may involve some type of conflicts and social problems, not only with teachers but also especially with peers (Hughes & Cao, 2018; Wong, 2015).

During early adolescence, peer relationships play a crucial role in students' lives and have a central role in shaping their identity and psychological well‐being (Rose & Rudolph) – but the relationships among students are influenced by various factors. For instance, a student's *gender* plays a particularly important role in shaping their social relationships (Kågesten et al., 2016). While most students (both girls and boys) prefer same‐gender friendships during early adolescence (Al‐Attar et al., 2017), girls often prioritize emotional closeness and intimacy in friendships, while boys focus more on sociability and practical support (Raufelder et al., 2010). Girls' networks tend to be smaller and more cooperative, whereas boys' networks are larger, more flexible and more competitive (Raufelder et al., 2010).

Furthermore, since communication is a crucial component of strong social relationships, *immigration background* (whether or not German is their mother tongue) may also play an important role in the quality of students' peer relationships in school. In particular, if students do not share the same native language as their peers, it could create a barrier that hinders the development of close relationships. This is affirmed by recent research findings which show that students with a migration background internalize problems rather than expressing them to their peers (Gutmann et al., 2019); have more experiences of peer victimization (Di Tata et al., 2024); and report more social problems (Hascher & Hagenauer, 2020) than other students. This may be the reason for the findings of Dežan and Sedmak (2023), who showed that migrant adolescents tend to be more satisfied with relationships with their teachers than those with their peers.

Another individual factor that might be decisive for peer relationships is the *educational track* the students are in (higher, medium or lower track schools in Germany). Research indicates that students placed in higher academic tracks often experience fewer social problems with peers, which in turn enhances their well‐being (Hascher & Hagenauer, 2020).

#### The teacher–student relationship

The teacher–student relationship evolves throughout a longer time period – for example, one school year – and is shaped by a multitude of social interactions, attitudes and emotions (Hamre & Pianta, 2006; Schlesier, Raufelder, & Moschner, 2023). Research studies conceptualize the teacher–student relationship primarily by characterizing a positive teacher–student relationship on the one hand, and a negative teacher–student relationship on the other. A positive teacher–student relationship is typically marked by low conflict potential, (perceived) warmth, trust, care, emotional security, support, closeness and fairness (Aldrup et al., 2018; Davis, 2003; Roorda et al., 2011). Conversely, a negative relationship is characterized by increased conflict potential, emotional distance and a lack of trust (Raufelder et al., 2016; Roeser & Eccles, 1998).

The relationship between students and teachers encompasses *all* social interactions and thus includes those interactions that are not related only to student emotions, but also to instruction and classroom management (Hamre & Pianta, 2010; Pianta et al., 2008). Nevertheless, the majority of research studies usually consider only the emotional or socio‐emotional aspects of the relationship – especially the aspects of emotional warmth or support, and conflict (Aldrup et al., 2018; Prewett et al., 2019; Roorda et al., 2011). However, according to the Prosocial Classroom Model (Jennings & Greenberg, 2009) or the CLASS instrument (Classroom Assessment Scoring System) (Pianta et al., 2008), classroom organization (behaviour management, etc.) and instructional support (e.g., feedback, etc.) are also part of social interactions in the classroom, alongside emotional support. This is supported by the fact that, considering it the other way around, the basic dimensions of instructional quality (i.e., classroom management, supportive climate and cognitive activation) (Dorfner et al., 2018) are closely connected to social relations in the classroom (Klieme, 2006) – a finding that is underpinned by empirical results (Longobardi et al., 2021).

The quality of teacher–student relationships is intertwined with the teacher's abilities regarding classroom management (time management, time on task, monitoring of learning time), which includes their efforts to “create an environment that supports and facilitates both academic and social‐emotional learning” (Evertson & Weinstein, 2006, p. 4). These skills in classroom management work together with the teacher's capacity to develop and maintain positive relationships with their students – as well as to foster positive peer interactions – all of which contribute to the development of positive social relationships in the classroom (Fauth et al., 2019; Hattie, 2023; Seidel & Shavelson, 2007).

The quality of teacher–student relationships is not static, but instead such characteristics change during the course of a student's primary and secondary school career. Particularly around the transition from primary to secondary school, teacher–student relationships in the lower grades are characterized by more closeness and warmth and also less conflict (Spilt et al., 2012; Wu & Hughes, 2015). Further, a decline in warmth and a high conflict potential in higher grades are closely linked to underachievement and low engagement, especially during the post‐transition phase (Hughes & Cao, 2018; Spilt et al., 2012). The transition from primary to secondary school coincides with a notable diminution in warmth, which surpasses the typical decline observed before or after the transition (Hughes & Cao, 2018); according to those authors, this decrease is over three times steeper than the usual yearly decrease preceding the transition. Since this decline is *not* linked to the specific grade of transition but to the transition process itself, Hughes and Cao (2018) assume that it stems from changes in the schooling environment, rather than from inherent developmental changes in the students. This assumption is supported by Schlesier et al. (2024) who showed that students have a desire for teacher support and teacher praise when feeling emotionally challenged – and this is higher in students who are directly in the transition phase from primary to secondary school, compared to students in higher grades of lower secondary school (Schlesier et al., 2024).

Moreover, differences concerning *gender* or *immigration background* have – consistently with the quality of peer relationships – already been identified in several studies. Although the overall trajectories for boys and girls are quite similar, boys report significantly less warmth and more conflict with their teachers than girls (Hughes & Cao, 2018; Spilt et al., 2012). In addition, there are initial indications that children with an immigration background also have more conflictual relationships with their teachers than children with a local background (Spilt et al., 2012).

#### Theoretical framework – Why and how the social motivation approach encompasses students' social relationships both with their peers and their teachers

Social relationships are complex and dynamic – and individuals have different relationships with the different people who surround them. This seems to be particularly complex in the school context, since students have relationships with different peers and different teachers, all of which take on a different nature. Even for peer relationships in particular, it is very difficult to integrate all the relationships of one student with their surrounding peers into one model that includes the dynamic, constantly reforming character of peer relationships, which are the result of daily interactions and group dynamics (Schlesier & Raufelder, 2025). However, there are several theories that can be used as a foundation for empirical studies focusing on peer relationships, such as social learning theory (Bandura, 1977); theories on developmental sequences in groups (König & Schattenhofer, 2022; Tuckman, 1965); theories about individual differences (Dweck et al., 1993); and theories on socio‐cultural approaches (Tudge, 1990), to name but a few (Schlesier & Raufelder, 2025). The fact that peer relationships in the classroom are extremely dynamic (especially around school transition phases during early adolescence) may be the reason why studies investigating relationships in the classroom often focus primarily on the teacher–student relationship.

Nevertheless, when considering the quality of students' relationships in their school environment, peer relationships should clearly be taken into account as, alongside students' relationships with teachers, these form an integral part of their school experiences and lives. As students' peer relationships and their relationships with teachers are closely intertwined – with each influencing the other in important ways (Hendrickx et al., 2017; Hughes et al., 2006), one approach could be to integrate students' peer relations into an existing theory of social relationships with teachers.

One of the most popular existing theories that could also capture the nature of peer relationships is the Attachment Theory (Bowlby, 1988). This theory proposes that – in accordance with the parent–child relationship – a nurturing connection with a teacher fosters a child's emotional stability and confidence (Davis, 2003). This, in turn, provides young students with the resilience they need to explore their surroundings actively and handle academic and social pressures more adeptly (Hughes & Cao, 2018; Pianta & Steinberg, 1992; Verschueren & Koomen, 2012). Riley (2011) highlights that emotional bonds between teachers and students often extend beyond the rational mind, fulfilling needs for belonging, growth and development. However, even though peers can sometimes offer similar emotional stability – particularly for students who lack family support – this is likely not the case for most students. Therefore, a different theoretical approach might be more suitable for integrating both types of students' social relationships in the classroom.

One approach that has been actively researched in recent years and has become established in educational (psychological) research is the Social Motivation Approach. This approach is based on the assumption that a supportive teacher–student relationship provides a sense of school belonging, as well as being and feeling valued at school – both of which pave the way to greater and more purposeful evidence of motivation and school engagement (Hughes et al., 2008; Klem & Connell, 2004; Skinner et al., 2008). Hughes and Cao (2018) point out that research involving adolescents refers predominantly to Deci and Ryan's (2002), Ryan & Deci (2017) self‐determination theory, which assumes that the fulfilment of students' basic needs in the classroom (i.e., social relatedness, perceived autonomy and felt competence) engenders their identification with school and thus their effort and engagement in learning and the school environment (Wigfield et al., 2015; Wigfield & Eccles, 2000). In other words, this approach posits that students of teachers who foster a sense of social relatedness, express confidence in their abilities and provide opportunities for autonomous learning within the classroom are more motivated at school. This claim has been confirmed in a meta‐analysis (Roorda et al., 2011), which reveals that positive teacher–student relationships are associated with higher student engagement and achievement, while the effects are correspondingly negative for negative relationships.

From the perspective of the Social Motivational Approach, a good relationship encompasses three basic needs within two key components: a primarily emotional–social component (need for social relatedness) and a primarily instructional component (comprising both the need to experience competence and the need for autonomy). This approach originally referred primarily to teacher–student relationships, but it can also be transferred to peers. As explained above, social relatedness is a central concept influencing motivation – and peer relationships are fundamental to students' sense of belonging at school (Knifsend et al., 2018; Liem & Martin, 2011). Students who feel accepted and valued by their peers are more motivated to engage in school (Liem & Martin, 2011).

But at the same time, meeting the different needs of students makes all social relationships in school inherently very complex. For example, teachers can promote social integration by implementing class team building events or rituals like celebrating students' birthdays, but the relationship between teacher and student can still be strained if the student does not have the opportunity to work independently or does not feel valued in terms of their competence level (e.g., when a teacher constructively criticizes a student's poor performance). Both components of the teacher–student relationship – the emotional and instructional components – must therefore be considered.

The Social Motivational Approach clearly indicates that social relationships (with both peers and teachers) are directly interwoven with students' levels of motivation. Thus, the following section provides an overview of the current state of research on students' relationships in school and their levels of motivation.

### The interplay of students' social relationships in school with motivation

Students' academic motivation – which refers to their beliefs, values and goal orientations in classroom contexts (Lazarides & Raufelder, 2017; Wentzel & Wigfield, 2009) – is important for numerous academic outcomes, such as school well‐being (Grigoryeva & Shamionov, 2014) and academic success (Lazarides et al., 2018). It is therefore hardly surprising that students' academic motivation is linked to their social relationships in academic contexts: peers and teachers can serve as important social associates and, as such, contribute to fulfilling basic needs for social relatedness and emotional security in school, as explained theoretically in the previous section (Juvonen & Wentzel, 1996; Rubin et al., 2009). In particular, positive relationships between students and their teachers and peers are associated with higher (intrinsic) motivation and more school engagement (Burns et al., 2022; Roorda et al., 2011; Wentzel, 2017). Conversely, negative relationships can be associated with lower motivation and engagement (Kindermann, 1993). Students who are victims of bullying have significantly lower academic motivation and experience less fulfilment of their basic needs of autonomy and competence (Young‐Jones et al., 2015). The variable‐centred study by Jiang and Yang (2022) with middle school students implies that teacher–student relationships are more meaningful for student motivation than peer relations, and also that positive relationships are more decisive for student motivation than negative relationships.

However, the findings cited are based primarily on variable‐centred approaches and are therefore unable to reveal individual differences (Roorda et al., 2011). But particular individual patterns of relationship quality could provide deeper insights into social structures in classrooms and could promote our understanding of links with student motivation. This would make a person‐centred approach valuable when considering students' social relationships at school together with their levels of motivation. Currently, studies in person‐centred approaches in the field refer mainly to motivational profiles and not to relationship profiles (Raufelder et al., 2022) – or to the synthesis of relationships and motivation (Raufelder et al., 2013). For example, studies using the framework of Raufelder et al. (2013) found four motivation‐relationship types in 7th and 8th graders: teacher‐dependent, peer‐dependent, teacher‐ and peer‐dependent, and teacher‐ and peer‐independent motivation types (Hoferichter et al., 2014, 2019; Jagenow, 2021; Jagenow et al., 2014, 2015; Raufelder et al., 2013). The peer‐dependent motivation type is evident for the largest group, containing over one‐third of the students in the sample, which underlines the importance of peer relations for student motivation but contradicts the findings of Jiang and Yang (2022) mentioned above. The teacher‐dependent motivation group is the smallest group, with one in ten students being categorized as belonging to this group (Raufelder et al., 2013). This means that the findings of variable‐centred and person‐centred approaches thus seem to produce somewhat contradictory results, indicating the need for more evidence in person‐centred approaches in order to gain deeper insight into the interconnectedness of students' social relationships at school and their motivation.

Regarding these four relationship‐motivation profiles, it is also known that most students tend to remain within the same profile (55.9%) during middle adolescence and that their assignment to a relationship‐motivation profile over time becomes less dependent on peers' influence on motivation and more dependent on that of teachers (Jagenow et al., 2015). The authors interpret this finding in terms of peers becoming less relevant for student motivation during middle adolescence, while the role of teachers becomes more meaningful. However, these results refer to a very specific period between 7th and 10th grade in secondary school and thus cannot be generalized to early adolescence, nor to the period of adjustment following the transition to secondary school.

This issue, where findings from a sample of significantly older children are applicable but not evident for the early adolescence period, also arises in the context of profiles regarding social relationships at school and their links to student motivation. For example, Burns et al. (2022) found four distinct profiles of teacher–student relationships for 9th and 10th graders, by means of latent profile analysis. They identified a positive teacher–student relationship (high socio‐emotional and instructional support, low conflict), a distant teacher–student relationship (medium to high socio‐emotional and instructional support, medium to low conflict), a complicated teacher–student relationship (high socio‐emotional and instructional support, but also high conflict) and a negative teacher–student relationship profile (low socio‐emotional and instructional support, high conflict). They also investigated differences between the profiles concerning student motivation (in terms of self‐efficacy, intrinsic value, utility value and cost). It has been demonstrated that students in the negative teacher–student relationship profile reported the lowest self‐efficacy, intrinsic value and utility value as well as the highest costs, while students in the positive and the complicated teacher–student relationship profiles reported most favourable motivation indicators (Burns et al., 2022). The same researchers also showed that students in the positive teacher–student relationship profile had the highest mean values for self‐efficacy, but those in the complicated profile reported the highest scores for intrinsic value and utility value. The distant teacher–student relationship profile falls between the positive and negative profiles in terms of reported motivation dimensions. Burns et al. (2022) also point out that supportive teacher–student relationships were more relevant to the students' adaptive motivation dimension (i.e., self‐efficacy) than conflict.

## AIMS AND HYPOTHESES

Research on students' social relationships in school has used mostly variable‐centred approaches and rarely focuses on individual relationship patterns. This has already generated important findings, such as the fact that students' social relationships are directly linked to their levels of engagement and motivation (Roorda et al., 2011) – which is in line with the assumption of the Social Motivation Approach (Deci & Ryan, 2014; Ryan & Deci, 2000, 2017; Wigfield et al., 2015; Wigfield & Eccles, 2000). However, only a few studies consider different social relationships using a person‐centred approach alongside motivation – they often mix these aspects or ignore the crucial phase of transition from primary to secondary school. Not least, while socio‐emotional components of teacher–student relationships have been extensively examined in prior research, the more structural or instructional components as part of the teacher–student relationship have received comparatively less attention. By focusing on these aspects, our study aims to offer a broader and more context‐sensitive understanding of how relationships are experienced in classrooms.

For these reasons, this study aimed

- To identify latent relationship quality profiles of students with their teachers and peers,
- To investigate differences in students' level of motivation in the identified relationship profiles,
- To explore the stability of these profiles (latent transitions) during the first year in secondary education (post‐transition phase) and
- To examine the role of students' motivation, gender, immigration background and educational track regarding the initial probabilities of being assigned to one of those profiles and transition probabilities between these profiles in the course of the first year in secondary education.

Previous research findings have already shown that students' relationships in class are closely related to their level of motivation (Raufelder et al., 2013; Wentzel, 2017). We expect to identify four profiles in total: one profile that demonstrates high quality of social relationships and another profile showing low quality of social relationships (Hypothesis H1a); and – in line with Raufelder et al. (2013) – we expect to find a profile with good teacher–student relationships, but poor peer relationships, and another profile the other way around (H1b).

Further, we assume that students with a higher perceived relationship quality report higher levels of intrinsic motivation, as intrinsically motivated individuals are more likely to seek relationships based on mutual understanding, shared interests and authentic interactions (Roorda et al., 2011) (H2a). Regarding extrinsic motivation, we assume the other way around, namely that students are in lower social quality profiles because, although they place value on meeting the expectations of those around them, this is unlikely to contribute to the development of a solid social network (H2b). We also expect the profiles to be relatively stable, as social relationships cannot be viewed as situational short‐term interactions but rather as a construct that develops over a period of time (H3). We assume that gender, immigration background (Jiang & Yang, 2022; Spilt et al., 2012) and educational track will influence profile affiliation over time, in that girls, students without an immigration background and those who are attending the higher educational track belong to profiles that demonstrate more positive perceptions of social relationships (H4a) (Hughes & Cao, 2018; Spilt et al., 2012).

Finally, we assume that students' level of intrinsic motivation influences the transition probabilities between the profiles. We anticipate – based on the results of Burns et al. (2022) – that students who display higher levels of intrinsic motivation more often transit to unfavourable profiles (i.e., worse relationship quality) or a complicated social relationship quality profile (for example, high perceived clarity and structure by the teacher, but low perceived student orientation and problems with peers), as these students are potentially driven more by their own interests and less by the expectations of those around them (H4b).

## METHODS

### Design and sample

The data were collected in a research study carried out from 2017 to 2019 with three time measurement points, at 22 Catholic schools in southern Germany. In total, *N* = 1343 students were surveyed online using valid and reliable questionnaires, at the beginning of year 5 (first year in secondary school; October–November 2017); towards the middle of year 5 (April–June 2018); and at the beginning of year 6 (second year in secondary school; October–November 2018). The surveys were conducted in the schools' computer rooms under the supervision of the teachers. Written informed consent was obtained from all parents before the surveys were conducted. Information was provided about the voluntary nature of participation, the possibility of withdrawing from participation and deleting the data, as well as the preservation of anonymity through pseudonymized data collection, evaluation and publication.

The age of the 1343 students ranged from nine to eleven years (*M* = 10.19, *SD* = .48) at the time of the first survey, and 80.7% were female. The high proportion of female students is due to the fact that almost half of the girls in the sample attend girls‐only schools. Around a quarter of the students (25.7%) had an immigration background (either the children themselves or at least one parent was born outside Germany).

### Instruments

#### Quality of social relationships in class

We tried to map as many levels and facets of relationship quality in the classroom as possible and to comprehensively record the teacher–student relationship and student–student relationships. Both emotional and instructional aspects were included in our questionnaire. As there is not yet a comprehensive questionnaire instrument for teacher–student and student–student relationships, various standardized subscales from different questionnaires were combined: The students' perceived quality of teacher–student and student–student relationships was assessed using four of the five dimensions of the EMU questionnaire (Helmke et al., 2016; Lenske, 2013): *Student orientation* includes six positive items regarding the teachers' feedback culture (“The teacher supports us by giving hints.”), emotional support by the teacher (“The teacher gives enough praise in class.”), as well as peer relationships (“We students are friendly to each other.”). Internal consistencies for this dimension were good (Cronbach's *α* = .86 ≤ *α* ≤ .88, McDonald's *ω* = .88 ≤ *ω* ≤ .90). *Clarity and structure of the teachers' instruction* is measured using five items which focus on the pace and clarity of teaching, and student understanding (“The teacher explains so that I can follow along well.”) (Cronbach's *α* = .83 ≤ *α* ≤ .85, McDonald's *ω* = .85 ≤ *ω* ≤ .87). The *activation* of the students by the lessons includes six items representing cognitive activation (“There are questions or tasks in class that really make me think.”) and collaboration in class (“I say something about the respective lesson topic.”) (Cronbach's *α* = .69 ≤ *α* ≤ .72, McDonald's *ω* = .71 ≤ *ω* ≤ .76). The subscale *classroom management* assesses whether the students can learn uninterrupted in class and consists of five positively formulated items (“I can work undisturbed during lessons.”) (Cronbach's *α* = .72 ≤ *α* ≤ .85, McDonald's *ω* = .79 ≤ *ω* ≤ .83).

All dimensions used to assess the perceived quality of teacher–student and student–student relationships were tested with one confirmatory factor analysis (CFA) per time measurement point (see Appendix S3) and reached a sufficient level of model fit (CFI = .89 ≤ *α* ≤ .92, TLI = .87 ≤ *α* ≤ .91, RMSEA = .05 ≤ *α* ≤ .067). School is a social space in which diverse socio‐emotional experiences are made. Social situations (whether positive or negative) generally have a strong emotional impact and are memorable (Schlesier & Raufelder, 2025). Accordingly, it is essential to capture such experiences in a comprehensive manner. It can be assumed that, when responding to our items, students predominantly recall particularly salient positive and negative experiences with both teachers and peers. By assessing students' social relationships at a more generalized level, we aim to obtain a broad picture of their perceived social relatedness within the school context as a whole. This perception of social relatedness, understood as a fundamental psychological need (Deci & Ryan, 2014), is closely associated with students' motivation. As we wanted to gain a comprehensive and global assessment of the students' relationship with their teachers, we asked them to think about all their teachers, and their lessons and interactions over the preceding few weeks, rather than just one teacher. Of course, this does not allow us to analyse subject‐specific or individual‐related information, but only to draw conclusions about more global perceptions of the teacher–student relationship and climatic aspects of the classes and the school. The quality of peer relationships was also captured using the sub‐dimension *absence of social problems in school* of the well‐being‐in‐school scale (Hascher, 2004). Using five inverse items (example: “Have you had any problems with your classmates in the last few weeks?”), the students were asked if they had experienced problems with their classmates or felt excluded or annoyed. The reliability of this scale was high at each measurement point (Cronbach's *α* = .83 ≤ *α* ≤ .86, McDonald's *ω* = .86 ≤ *ω* ≤ .88). The answer format for all dimensions ranged from 1 = *do not agree at all*, to 5 = *fully agree*; the inverse scale regarding peer relationships was recoded in such a way that high values also indicate positive relationships with peers.

#### Student motivation

The students' levels of extrinsic and intrinsic learning motivation (respectively, EM and IM) (Ryan & Connell, 1989) were assessed as follows: EM was measured using items regarding extrinsic external regulation (five items, example: “I learn because I have to and not because I want to.”; Cronbach's *α* = .74, McDonald's *ω* = .81), which reflects the amount to which students are motivated by rewards and reinforcement or to avoid penalty. IM was measured by three items, for example: “I learn because it's fun.”; Cronbach's *α* = .88, McDonald's *ω* = .87), which reflects the students' willingness to learn because they are interested in the learning process itself or in the learning content. The CFA conducted (see Output C1, Appendix S3) confirms the two‐factor structure, since it shows a good/excellent fit of the models for each time measurement point (CFI = .94 ≤ *α* ≤ .96, TLI = .92 ≤ *α* ≤ .94, RMSEA = .10 ≤ *α* ≤ .07).

#### Covariates

Since student motivation and the perception of social relationships both depend on students' gender, educational track attained or immigration background, we included those variables as covariates. The covariates included were dichotomous, that is, gender (1 = male/0 = female); educational track (1 = higher track/0 = medium track); immigration background (1 = no immigration background/0 = immigration background).

### Data analysis procedures

First, descriptive analyses were conducted in order to explore the mean values, standard deviations, development over time (repeated measure ANOVA) and correlations.

Missing values were analysed using Little's MCAR test and were found to be completely at random for t1 and t2, but not for t3 (t1: *χ* ^2^(74) = 82.24, *p* = .24; t2: *χ* ^2^(86) = 92.72, *p* = .29; t3: *χ* ^2^(85) = 171.36, *p* < .001). As missing data at t3 were not completely at random, logistic regression analyses were conducted to explore patterns of missingness. Girls showed a higher likelihood of missing values, while students with an immigration background showed a slightly lower likelihood of missing values (see Output D1, Appendix S4), which should be considered a limitation.

Given the nested data structure (students within schools), intraclass correlation coefficients (ICCs) were calculated for the variables representing social relationships at school (Table 1). Values greater than .05 indicate that group affiliation should be taken into account (LeBreton & Senter, 2008). As ICCs exceeded .05 only for the estimation of classroom management (see Table 1), we refrained from conducting multilevel latent profile analyses (LPAs).

**TABLE 1:** Means (standard deviations) and missing values of the variables for each time measurement point.

Variable	T1		ICC	T2		ICC	T3		ICC	Repeated measures ANOVA
	Mean (SD)	Missings^a^, ^b^		Mean (SD)	Missings^a^, ^c^		Mean (SD)	Missings^a^, ^d^		
Ed. Track^e^		13.67%								
Gender^e^		15.08%								
Immigr.^e^		13.74%								
EM	2.61 (.91)	16.79%		2.51 (.89)	9.88%		2.51 (.86)	22.81%		*F*(2, 717) = 3.23, *p* < .05, $\eta_{p}^{2}=.01$
IM	3.02 (1.02)	18.42%		2.84 (1.04)	10.85%		2.62 (1.02)	23.03%		*F*(2, 693) = 44.47, *p* < .001, $\eta_{p}^{2}=.11$
SO	4.02 (.73)	27.04%	.02	3.90 (.77)	16.20%	.06	3.69 (.83)	23.70%	.04	*F*(2, 601) = 46.38, *p* < .001, $\eta_{p}^{2}=.13$
CM	3.61 (.72)	25.04%	.06	3.48 (.69)	14.64%	.06	3.30 (.76)	23.11%	.06	*F*(2, 627) = 40.02, *p* < .001, $\eta_{p}^{2}=.11$
CS	3.88 (.71)	28.90%	.01	3.72 (.75)	17.61%	.03	3.61 (.77)	24.52%	.01	*F*(2, 576) = 29.38, *p* < .001, $\eta_{p}^{2}=.09$
ACT	3.48 (.57)	29.12%	.00	3.41 (.57)	18.13%	.05	3.31 (.59)	24.37%	.00	*F*(2, 583) = 16.01, *p* < .001, $\eta_{p}^{2}=.05$
NSP	4.30 (.82)	23.33%	.04	4.18 (.90)	12.70%	.01	4.09 (.95)	24.37%	.02	*F*(2, 647) = 12.52, *p* < .001, $\eta_{p}^{2}=.04$

Abbreviations: Ed. Track, Educational track; Immigr., Immigration background; ACT, activation during the lessons; CM, classroom management; CS, clarity and structure; EM, extrinsic motivation; ICC, intraclass correlation; IM, intrinsic motivation; NSP, Absence of social problems with peers; SO, student orientation of the teachers; T, time point.

^a^ Measured in percent.

^b^ MCAR: *χ* ^2^(74) = 82.24, *p* = .24.

^c^ MCAR: *χ* ^2^(86) = 92.72, *p* = .29.

^d^ MCAR: *χ* ^2^(85) = 171.36, *p* < .001.

^e^ Captured at t1.

Following the recommendation for conducting latent transition analyses (LTAs) (Nylund et al., 2007; Sorgente et al., 2019; Vermunt, 2010), a multistep procedure was then performed.

The constructs included in our analyses were first explored descriptively and checked with regard to their longitudinal measurement invariance as well as their measurement invariance with regard to subgroups (gender and immigration background, Tables B2 and B3 in Appendix S2): Configural, metric, scalar and strict longitudinal measurement invariance for all categorical data and all constructs were tested (Liu et al., 2017) using the R package *lavaan* (version 0.6‐8) (Rosseel, 2012). The measurement invariance test provides information on whether the scales used depict the same construct with the same metric at all three time measurement points or for different subgroups within the sample. For testing longitudinal measurement invariance, we used the diagonally weighted least squares (DWLS) estimator, as it provides robust estimates standardized for the mean and variance. The quality of the models was assessed using the two‐strategy approach (Chen, 2007), which postulates an acceptable fit if a combination of CFI ≥ .95/.90 and RMSEA values ≤ .05/.06 is given (Hu & Bentler, 1999). Additionally, Chen's (2007) cut‐off criteria were used to assess measurement invariance between groups: a change in CFI of ≤.010 and a change in RMSEA of ≤.015 were considered acceptable. Strong or even strict longitudinal measurement invariance, as well as measurement invariance across groups, was confirmed for nearly all constructs (see Appendix S2). Measurement invariance with regard to subgroups within the sample was tested using the MLR estimator, which is robust to non‐independent observations (Rosseel, 2017). Missing values ranging from 10% to 29% (see Table 1) were replaced by applying the full information likelihood method (Graham, 2009). Further information regarding the different levels of longitudinal measurement invariance and measurement invariance for subgroups is displayed in Tables B1–B3 (Appendix S2).

Thereafter, latent profile analyses (LPAs) were conducted for each time measurement point. LPA is a person‐centred approach that is used for the explorative investigation of different profiles within datasets based on latent features of the persons in the sample (Hickendorff et al., 2018). Data regarding the students' perceptions of social relationships (including their teacher–student relationship and peer relationships) were used to cluster the data at each time measurement point. The model with equal variances and fixed variances (EEI) was chosen to estimate different profile solutions, since it is the most parsimonious (Rosenberg et al., 2018). The different profile solutions were compared using measures of relative fit (*Bayesian Information Criterion (BIC) and Akaike Information Criterion (AIC)*), with lower values indicating a better fit (Ferguson et al., 2020). The BIC is a conservative measure that prefers parsimonious models, while the AIC does not assume such strong parsimony constraints (Ferguson et al., 2020). A bootstrap likelihood ratio test (BLRT) was conducted for each LPA in order to compare the fit of the model with a model that contained one less profile (Ferguson et al., 2020; Nylund et al., 2007). Additionally, *entropy* – which ranges between 0 (low quality of posterior classification of the individuals in the sample) and 1 (perfect posterior classification) – was interpreted as a relative measure of the classification (Ferguson et al., 2020; Sorgente et al., 2019). Entropy values greater than .70 can be interpreted as adequate (Fonseca & Cardoso, 2007). In addition to the consideration of different fit indices when selecting the appropriate number of profiles, the content and theoretical interpretability of the profiles found are also important factors. With a sufficient sample size, even smaller profiles (<30) can provide substantial information about underlying latent patterns. In our case, it can theoretically be assumed that only very few students have low scores on all variables (Ferguson et al., 2020; Marsh et al., 2009; Vincent & Weir, 2012). The certainty of classification was assessed using information on the minimum (prob. min) and maximum (prob. max) of the diagonal of the average latent class probabilities for the most likely class membership, both of which should be as high as possible (Jung & Wickrama, 2008). Additionally, the several profile solutions were compared according to their interpretability. Thus, we chose the models with the most favourable combination of AIC, BIC, entropy and minimum/maximum average latent class probabilities, which was also the most interpretable in terms of content (Ferguson et al., 2020). Missing values were replaced via the random forest algorithm (Breiman, 2001), using the package *missForest* (version 4.6–14) (Stekhoven & Bühlmann, 2012).

After exploration of the LPAs for each time measurement point and selection of the most suitable profile solution, latent transition models were calculated (Hickendorff et al., 2018). Latent transition analysis enables the identification of subgroups within a sample that are caused by latent characteristics and their analysis over time. Heterogeneity in the sample – at within and between levels, as well as the quantitative and qualitative differences in the study variables, was taken into account. Latent transition models contain autoregressive paths from one latent class variable at a later time measurement point to the same latent class variable at an earlier time measurement point. Thus, LTAs start with more than one latent class variable captured at different time measurement points and investigate the movement of individuals from one to another class between the measurement points (Sorgente et al., 2019). Transitions are expressed as probabilities that indicate the likelihood of changing (i.e., transitioning) from one latent status to another.

In this study, following the procedure proposed by Sorgente et al. (2019), firstly the latent transition model was specified without covariates (Model 1), and profile prevalence and transition probabilities were obtained in order to estimate profile affiliation over time. Secondly, observed dichotomous covariates that affect the initial probabilities of membership in a particular latent profile and the transition probabilities between the profiles were added (Model 2). Covariates included as predictors of membership in a latent profile were regressed on the latent profile in order to estimate its impact. Finally, a model with the dichotomous covariates affecting the initial probabilities and metric covariates affecting the transition probabilities (Model 3) was specified using the package *Lmest* (Bartolucci et al., 2017).

In addition to the regression of covariates predicting membership in a latent profile, further regressions of the covariates affecting transition probabilities were specified in the model (Sorgente et al., 2019). Bootstrapping that performed parametric resampling with 100 samples for each latent transition model (with and without covariates) was conducted to estimate standard errors. Logit transformations, that are a common tool for converting probabilities in linear relations, were interpreted in order to estimate the effects of covariates on initial and transition probabilities for the different profiles, over time. To obtain the actual probabilities, the logit values were back‐transformed using the following equation:

(1) $$p=\frac{e^{\text{logit}}}{1+e^{\text{logit}}}$$

Equation (1) is a formula for calculating the probability from log‐odds.

## RESULTS

### Descriptive statistics

The students' perceptions of their social relationships are relatively high, although decreasing slightly over time. In particular, *teachers' student orientation* and perceived *absence of social problems with classmates* are rated very positively by the students. The students display moderate levels of both intrinsic and extrinsic motivation, with the values of intrinsic motivation exceeding the values of extrinsic motivation at all measurement points. Although intrinsic motivation decreases steadily over time, the level of extrinsic motivation seems to be relatively stable (see Table 1).

### Correlations

The students' levels of intrinsic and extrinsic motivation are correlated with their perceptions of their social relationships in school. At measurement point t1, while the dimensions of teacher–student and student–student relationships are negatively correlated with extrinsic motivation on a low effect size level (t1: student orientation and extrinsic motivation: *r* = −.16, *p* < .001; absence of social problems and extrinsic motivation: *r* = −.25, *p* < .001), they are moderately and positively correlated with intrinsic motivation (t1: student orientation: *r* = .32, *p* < .001; clarity and structure: *r* = .43, *p* < .001) (see Table A1 in Appendix S1).

The same is the case for the correlations at t2, where all variables regarding teacher–student and student–student relationships are also negatively correlated with extrinsic motivation (t2: student orientation and extrinsic motivation: *r* = −.24, *p* < .001; absence of social problems: *r* = −.22, *p* < .001) and positively correlated with intrinsic motivation (t2: student orientation and intrinsic motivation: *r* = .33, *p* < .001; clarity and structure and intrinsic motivation: *r* = .36, *p* < .001) (see Table A2 in Appendix S1).

Finally, at t3 all variables regarding the social relationships of the students in class are negatively correlated with extrinsic motivation (t3: student orientation and extrinsic motivation: *r* = −.23, *p* < .001; absence of social problems: *r* = −.25, *p* < .001) and positively correlated with intrinsic motivation (t3: student orientation and intrinsic motivation: *r* = .34, *p* < .001; clarity and structure and intrinsic motivation: *r* = .37, *p* < .001) (see Table A3 in Appendix S1).

### Latent profile analyses (LPAs)

Latent profile analyses (LPAs) were calculated separately for the data at each time measurement point in order to confirm the existence of similar latent states for each measurement point. The comparison of the different profile solutions regarding comparative fit indices (AIC and BIC), comparative measures of the quality of the classification (entropy) and the classification certainty (prob. min and prob. max) reveals that the *four‐profile solution* is most suitable for all three time measurement points (see Table 2). Additionally, this solution provided the most interpretable profiles in terms of content (for reference, the output and means of the three‐profile solution are provided in Output E1, Appendix S5).

**TABLE 2:** Fit indices of several profile solutions calculated for each time measurement point.

Classes	AIC	BIC	Entropy	Prob_min	Prob_max	BLRT_p
t1						
2	17,433.97	17,517.24	.82	.93	.96	.01
3	17,287.71	17,402.22	.81	.79	.96	.01
**4**	**16,771.80**	**16,917.54**	.**81**	.**87**	.**92**	.**01**
5	16,959.07	17,135.04	.78	.54	.89	.31
t2						
2	1725.84	17,338.12	.81	.94	.95	.01
3	16,659.88	16,774.39	.81	.86	.93	.01
**4**	**16,400.98**	**16,546.72**	.**85**	.**85**	.**93**	.**01**
5	16,368.14	16,545.10	.78	.80	.89	.01
6	16,157.13	16,365.33	.81	.81	.90	.01
7	16,108.54	16,347.97	.76	.73	.88	.01
8	16,062.29	16,332.95	.77	.69	.90	.01
9	15,955.20	16,257.09	.77	.61	.98	.01
10	15,869.13	16,202.24	.79	.66	.98	.01
11	15,898.65	16,263.00	.78	.60	.98	.47
t3						
2	17,483.47	17,566.75	.77	.93	.94	.01
3	16,885.49	16,999.99	.86	.90	.94	.01
**4**	**16,479.76**	**16,625.50**	.**81**	.**88**	.**94**	.**01**
5	16,370.81	16,547.78	.78	.80	.96	.01
6	16,285.55	16,493.74	.82	.80	.98	.01
7	16,266.75	16,506.18	.75	.69	.98	.01
8	16,190.12	16,460.77	.75	.72	.95	.01
9	16,044.92	16,346.80	.79	.76	1.00	.01
10	16,030.55	16,363.66	.77	.69	1.00	.01
11	15,979.34	16,343.68	.77	.67	1.00	.01

*Note*: The solution with the best fit is highlighted in bold.

The four profiles, which can be interpreted well in terms of content characteristics, are of different sizes and are also characterized by differences in composition. Profile 4, which is the largest group, is characterized by comparably high means (but not the highest) in all dimensions of perceived social relationships, but especially in terms of the *absence of social problems* – this profile is labelled ‘solid quality of social relationships’. The profile comprises 41.3% of the students in the sample at t1, 47.1% at t2 and 41.8% at t3, and also includes the highest percentage of female students. Regarding the students' levels of intrinsic and extrinsic motivation, it was found that these students have high levels of *intrinsic motivation* and relatively low levels of *extrinsic motivation*.

The second largest profile (Profile 2) is labelled ‘moderate teacher relationship but problems with peers’ and includes 30.3% of the students at t1, 35% at t2 and 26.7% at t3. The profile is made up of the highest percentages of students in the medium educational track and those with an immigration background. Students characterized by moderate perceptions of their social relationships with teachers and peers are also moderately motivated. These students show relatively high values for *extrinsic motivation* and medium to low values for *intrinsic motivation*.

Profile 3 contains 26.1% of the students at t1, 14.6% at t2 and 18.9% at t3. It is characterized by overall high values for perceived relationship quality with both teachers and peers and is thus labelled ‘high quality of social relationships’. It contains a high percentage of female students and students in higher academic educational tracks, while the percentage of students with an immigration background is the lowest. These students report high levels of *intrinsic motivation* and low levels of *extrinsic motivation*. This profile is characterized by the highest values for *intrinsic motivation*, compared to all the other profiles.

**FIGURE 1:**
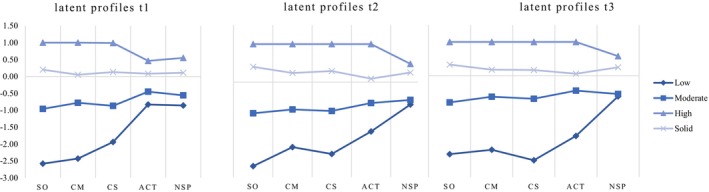
Four‐profile solution for each time measurement point. We used *z*‐standardized means for this analysis. ACT, activation during the lessons; CM, classroom management; CS, clarity and structure; NSP, absence of social problems with peers; SO, student orientation of the teachers.

**FIGURE 2:**
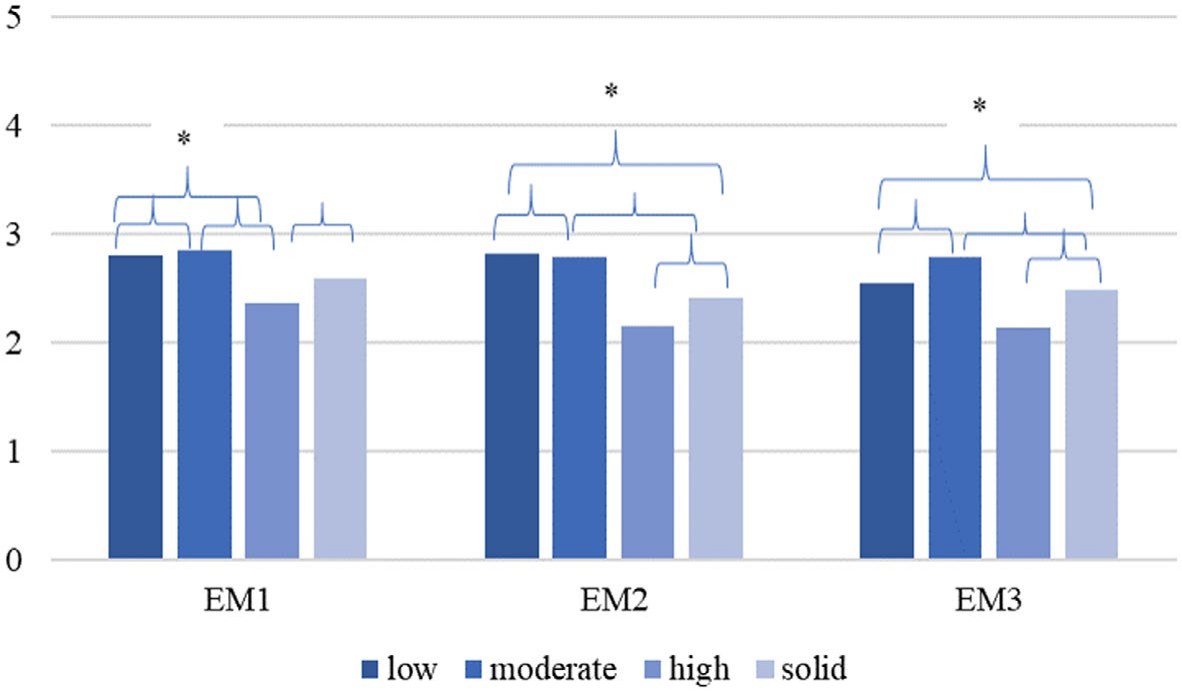
. Mean values for extrinsic motivation for each profile and time measurement point. EM1 to EM3: Extrinsic motivation per time measurement points 1–3. Bars that are connected with brackets display means that differ significantly.

**FIGURE 3:**
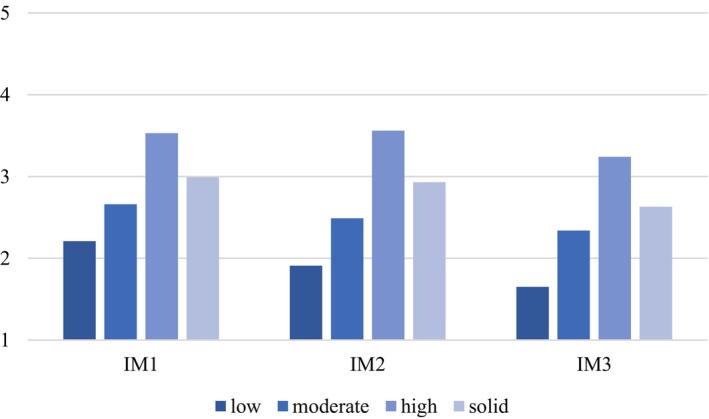
Mean values for intrinsic motivation for each profile and time measurement point. IM1 to IM3: Intrinsic motivation per time measurement points 1–3. All mean values of the several profiles differ significantly from one another.

The smallest profile (Profile 1) is labelled ‘low quality of social relationships’ and is distinguished by low values regarding most of the dimensions of social relationships with teachers and peers. The students in this profile (2% at t1, 3.5% at t2 and 2.8% at t3) report the lowest mean for the dimension of *absence of social problems*, which means that they experience a comparatively high level of social problems with their peers. This profile includes the highest percentage of students in academic educational tracks and male students, and also the second highest percentage of students with an immigration background. Students in the ‘low’ profile can be characterized by overall low motivation. In particular, their values for *intrinsic motivation* are comparably low (see Table 3, Figures 1, 2, 3).

**TABLE 3:** Mean values of the clustering variables and outcomes, and composition of the profiles for each time measurement point.

Profiles	*N*	Mean values of the clustering variables					Composition			Outcomes	
		SO^a^	CM^a^	CS^a^	ACT^a^	NSP^a^	Ed. track^b^	Gender^b^	IB^b^	EM^a^	IM^a^
t1											
Low (X1)	34	2.27	2.01	2.60	2.89	3.62	57.76	51.52	27.27	2.80	2.21
Moderate (X2)	407	3.36	3.08	3.29	3.08	3.88	37.79	78.82	28.20	2.84	2.66
High (X3)	350	4.72	4.32	4.52	3.56	4.72	56.15	81.70	21.77	2.36	3.53
Solid (X4)	555	4.14	3.53	3.95	3.35	4.38	50.64	83.41	26.34	2.59	2.99
t2											
Low (X1)	47	2.19	2.30	2.32	2.65	3.63	25	67.50	30.00	2.81	1.91
Moderate (X2)	196	3.25	2.98	3.15	3.08	3.77	46.25	77.02	28.64	2.79	2.49
High (X3)	470	4.80	4.40	4.68	4.11	4.63	58.79	79.01	23.64	2.15	3.56
Solid (X4)	633	4.18	3.63	3.91	3.44	4.38	48.90	84.96	23.71	2.40	2.93
t3											
Low (X1)	38	1.87	1.77	1.82	2.34	3.61	31.03	75.86	31.03	2.54	1.65
Moderate (X2)	495	3.03	2.85	3.10	3.06	3.57	44.53	77.89	29.26	2.79	2.34
High (X3)	254	4.60	4.10	4.51	3.83	4.58	51.75	80.36	23.35	2.14	3.24
Solid (X4)	561	3.92	3.40	3.69	3.32	4.29	51.17	83.23	29.39	2.48	2.63

Abbreviations: ACT, activation during the lessons; CM, classroom management; CS, clarity and structure; EM, extrinsic motivation; IM, intrinsic motivation; NSP, absence of social problems with peers; SO, student orientation of the teachers.

^a^ Unstandardized means.

^b^ Percentage of academic educational track (Ed. Track), females and students with an immigration background (IB).

The students in the various profiles differ significantly in their *extrinsic motivation* at t1, *F*(1, 1344) = 5.39, *p* = .02 and t3, *F*(1, 1344) = 33.71, *p* < .00. Tukey tests for multiple comparisons of the profiles at each time measurement point reveal significant differences in *extrinsic motivation* at t1 between profiles 2 and 1 (95% CI [.11, .39]), profiles 3 and 1 (95% CI [−.38, −.09]), profiles 3 and 2 (95% CI [−.64, −.33]) and profiles 4 and 3 (95% CI [.06, .83]). The mean differences between the profiles remain at t2 (2–1: 95% CI [.09, .73]; 3–1: 95% CI [.26, .52]; 4–1: 95% CI [−.43, −.08]; 4–2: 95% CI: [−1.01, −.32]; 4–3: 95% CI [−.83, −.47]); and at t3 (2–1: 95% CI [.18, .42]; 4–1: 95% CI [−.48, −.20]; 4–2: 95% CI [−.79, −.50]; 4–3: 95% CI [−.73, −.06]) (see Figure 2).

Group differences in *intrinsic motivation* due to profile affiliation can also be found between each profile at each of the three time measurement points (t1: *F*(1, 1344) = 20.04, *p* < .00), t2: (*F*(1, 1344) = 21.07, *p* < .00) and t3 (*F*(1, 1344) = 62.74, *p* < .00). According to the Tukey tests conducted, all profiles differ significantly from each other at t1 1 to 2: 95% CI [−.48, −.19], 3 to 1: 95% CI [.38, .69], 4 to 1: 95% CI [−1.18, −.58], 3 to 2: 95% CI [.71, 1.04], 4 to 2: 95% CI [−.85, −.04] and 4 to 3: 95% CI [−1.72, −.91]. The significant differences remain also at t2 (1–2: 95% CI [−1.32, −.60], 3–1: 95% CI [−.58, −.30], 4–1: 95% CI [−.44, −.83], 3–2: 95% CI [.16, .88], 4–2: 95% CI [1.21, 1.98] and 4–3: 95% CI [.87, 1.28]) and t3 (1–2: 95% CI [.43, .15], 3–1: 95% CI [−1.35, −.61], 4–1: 95% CI [.45, .77], 3–2: 95% CI [−1.07, −.31], 4–2: 95% CI [.73, 1.07] and 4–3: 95% CI [1.20, 1.97]) (see Figure 3).

### Latent transition analysis

Based on the four‐profile solution revealed by the LPAs, latent transition analysis with four latent states was conducted. Following the recommendation of Sorgente et al. (2019), three different models were specified, which increasingly consider covariates and therefore become more complex.

### Initial probabilities and stability of the profiles (model 1 without covariates)

In the first model (LogLik = −15,862.64, AIC = 31,849.28, BIC = 32,171.98) that did not contain covariates, the initial probabilities and transition probabilities were estimated. The initial probabilities of the four named profiles are 3%, 29%, 22% and 46% for the profiles characterized, respectively, by low, moderate, high and solid values on the cluster variables.

**FIGURE 4:**
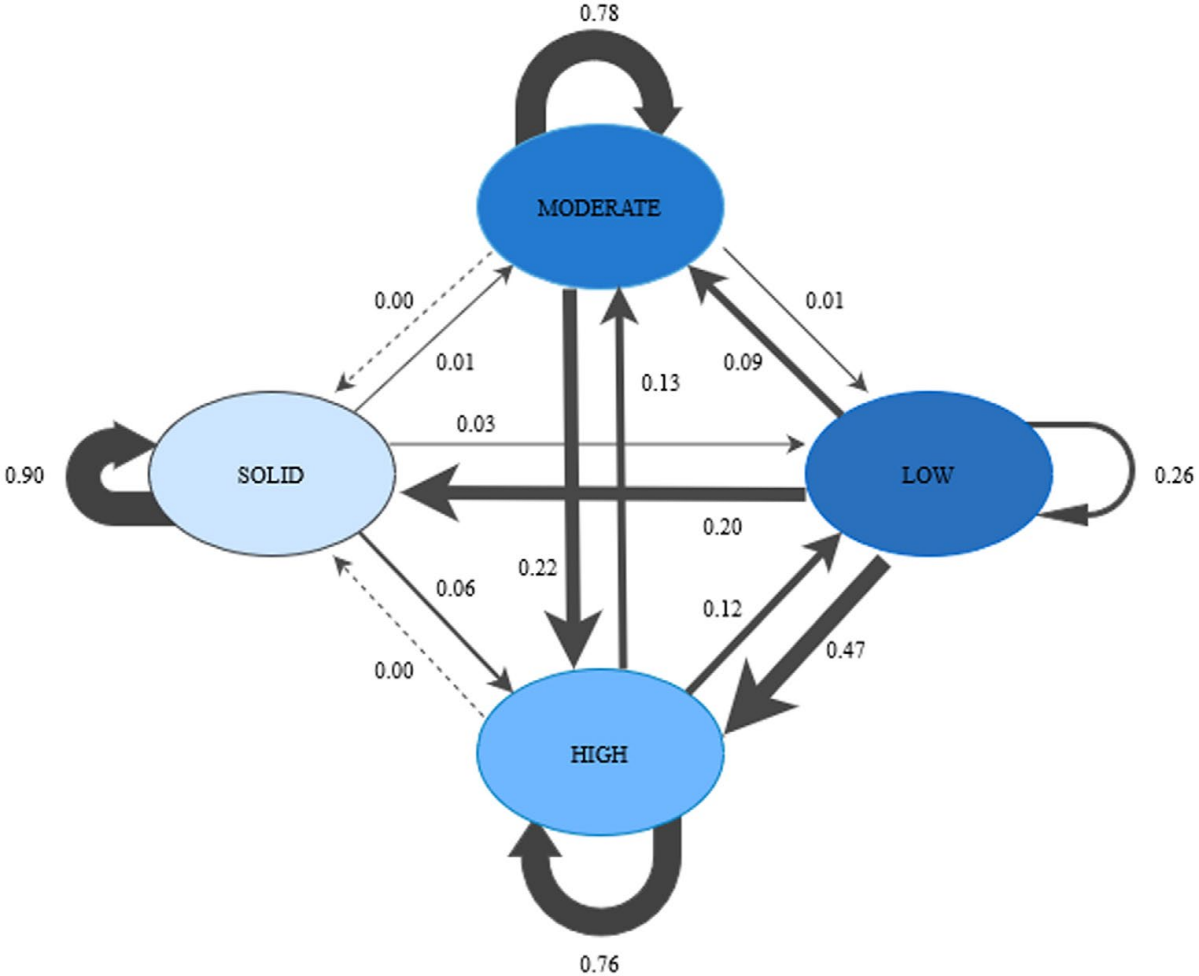
Transition probabilities between the profiles (T1 − T2 and T2 − T3). SOLID, solid quality of social relationships; MODERATE, moderate quality of social relationships; HIGH, high quality of social relationships; LOW, low quality of social relationships. The thickness of the arrows indicates the probability of transition: Thin arrows indicate a low probability, thick arrows a high probability, dotted arrows indicate that the transition is unlikely.

The largest profile (Profile 4, ‘solid’) seems to be very stable, since 93% (95% CI [.89, .97]) of the students remain there from t1 to t2 and 87% (95% CI [.83, .91]) of them remain from t1 to t3. The second largest profile, characterized by moderate values on all cluster variables (Profile 2, ‘moderate’), is also characterized by high stability: 76% (95% CI [.68, .84]) of the students remain there between t1 and t2 and 75% (95% CI [.69, .81]) remain from t1 to t3. The third largest profile, characterized by an overall high quality of social relationships (Profile 3, ‘high’), is relatively stable as 73% (95% CI [.65, .81]) of the students stay within the profile from t1 to t2 and 83% (95% CI [.77, .89]) of them stay there from t1 to t3. The smallest profile (Profile 1, ‘low’), with the lowest values regarding all cluster variables, is not stable at all: 25% (95% CI [.07, .43]) of the students stay in this profile from t1 to t2 and 26% (95% CI [.14, .38]) from t1 to t3. The mean probabilities are displayed in Figure 4. The size of the profiles, their stability and transition probabilities are displayed in Table 4.

**TABLE 4:** Distribution of students to the latent profiles and stability and transition probabilities of the profiles over time.

	Distribution to the profiles				Stability and transition probabilities							
					T1 − T2				T2 − T3			
	T1	T2	T3	Overall	X1	X2	X3	X4	X1	X2	X3	X4
	*N* (%)	*N* (%)	*N* (%)	*N* (%)								
Low (X1)	34 (2.5%)	47 (3.5%)	38 (2.8%)	40 (3%)	.**25 (.09)**	.08 (.08)	.44 (.10)	.23 (.09)	.**26 (.06)**	.09 (.06)	.50 (.07)	.16 (.07)
Moderate (X2)	407 (30.3%)	470 (35%)	493 (26.7%)	389 (29%)	.00 (.00)	.**73 (.04)**	.27 (.04)	.00 (.00)	.01 (.01)	.**83 (.03)**	.16 (.03)	.00 (.00)
High (X3)	350 (26.1%)	196 (14.6%)	254 (18.9%)	296 (22%)	.14 (.03)	.10 (.03)	.**76 (.04)**	.00 (.00)	.10 (.02)	.16 (.03)	.**75 (.03)**	.00 (.00)
Solid (X4)	555 (41.3%)	633 (47.1%)	561 (41.8%)	618 (46%)	.04 (.01)	.01 (.01)	.03 (.02)	.**93 (.02)**	.02 (.01)	.01 (.01)	.09 (.02)	.**87 (.02)**

*Note*: Stability values are highlighted in bold.

Abbreviations: X1, low quality of social relationships; X2, moderate quality of social relationships; X3, high quality of social relationships; X4, solid quality of social relationships.

#### Transitions from T1 to T2

The transitions from the smallest profile (‘low’) can be described as follows: Due to the low stability of Profile 1 (‘low’), 23% (95% CI [.05, .41]) of the students transit to the profile characterized by solid quality of social relationships (Profile 4) and 44% (95% CI [.24, .64]) transit to the profile with high values (Profile 3). Further, 8% (95% CI [−.08, .24]) of the students transit to the moderate profile (Profile 2). The transition from Profile 1 (‘low’) to Profile 2 (‘moderate’) is not significant.

Transitions from Profile 2 (‘moderate’) can be characterized as follows: 27% (95% CI [.19, .35]) of the students transit to Profile 3 (‘high’). All other transitions are not significant.

Transitions from Profile 3 (‘high’) show that 14% (95% CI [.08, .20]) of the students transit to the profile with the lowest values on all cluster variables (Profile 1), and a further 10% (95% CI [.04, .16]) of them transit to the moderate profile (Profile 2).

Concerning the profile characterized by solid values on all cluster variables (Profile 4), 4% (95% CI [.02, .06]) of the students transit to the low quality profile (Profile 1). All other transitions are not significant.

#### Transitions from T2 to T3

Regarding the transitions to t3, we observe that 16% (95% CI [.02, .30]) of the students who were formerly in the low profile (Profile 1) transit to the solid quality profile (Profile 4), while 50% (95% CI [.36, .64]) of them transit to the profile with high values on all cluster variables (Profile 3).

Transitions from the moderate profile (Profile 2) show that 16% (95% CI [.10, .22]) of the students transit to the profile with overall high values on all cluster variables (Profile 3).

Concerning the transition from the profile with high quality (Profile 3), students transit mostly to the profile characterized by moderate values (Profile 2) (16%, 95% CI [.10, .22]). Another 10% (95% CI [.06, .14]) of students transit to the profile with the lowest values on all cluster variables (Profile 1).

Students who were formerly in the profile with overall solid values on all cluster variables (Profile 4) change mostly (9%, 95% CI [.05, .13]) to the profile with high values (Profile 3) on all cluster variables. A further 2% (95% CI [.00, .04]) of students transit to the profile with the lowest values on all cluster variables (Profile 1).

#### Effects of the dichotomous covariates on the initial probabilities (model 2)

The second model (Loglik = −15,846.68, AIC = 31,883.35, BIC = 32,377.82) reveals how the dichotomous covariates (educational track, gender and immigration background) affect the logit for the initial probabilities between the profiles.

The profile with moderate values (Profile 2) is considered the reference category for the initial probability of belonging to profiles 1 (‘low’), 3 (‘high’) and 4 (‘solid’). The intercepts represent the odds of students being assigned to profiles 1, 3 and 4 (compared to Profile 2, ‘moderate’) when the dichotomous covariates are 0 (female gender, immigration background and medium educational track). The coefficients for gender, immigration background and educational track indicate by how much the log‐odds of the probability of allocation to one of profiles 1, 3 and 4 (compared to Profile 2) increase or decrease if the dichotomous variable increases by one unit. Thus, they display the changes in the case when students have male gender, no immigration background and attend the academic educational track.

The intercept for the assignment probability in Profile 1 (‘low’) is *B* = 1.73 (*SE* = .40; 95% CI [.96, 2.51]). It is reduced by *B* = −.56 (*SE* = .43; 95% CI [−1.40, .27]) units for male gender, by *B* = −.13 (*SE* = .42, 95% CI [−.96, .70]) units for no immigration background, and it is increased by *B* = .42 (*SE* = .41; 95% CI [−.37, 1.22]) units for attending the higher academic educational track. The influence of the covariates on the initial assignment probability is not significant.

Concerning the probability of classification in Profile 3 (‘high’), the intercept is *B* = 1.34 (*SE* = .39, 95% CI [.57, 2.10]), which means an initial probability of 79% in case all covariates are zero (female gender, immigration background and medium educational track). The intercept decreases by *B* = −1.04 (*SE* = .41; 95% CI [−1.85, −.23]) units for males. Further, it increases by *B* = .20 (*SE* = .41; 95% CI [−.61, 1.01]) units when no immigration background is given and by *B* = 1.01 (*SE* = .39; 95% CI [.25, 1.77]) units when the student attends the higher academic educational track. The influences of gender and educational track are significant. Male students are 26% less likely to be in this profile, while students attending the academic educational track are 73% more likely to be assigned to this profile.

In relation to the initial assignment probability for Profile 4 (‘solid’) compared to Profile 2 (‘moderate’), the intercept is *B* = 2.35 (*SE* = .37; 95% CI [1.63, 3.07]). In the case where all the predictors are zero (female gender, immigration background and medium educational track), the initial probability of being assigned to this profile (compared to Profile 2) is 91%. The intercept reduces by *B* = −.92 (*SE* = .39; 95% CI [−1.67, −.16]) units for males, but increases by *B* = .24 (*SE* = .39; 95% CI [−.53, 1.00]) units when there is no immigration background and increases by *B* = .64 (*SE* = .37, 95% CI [−.09, 1.36]) units when the student attends academic educational track. The influence of gender is significant: male students have a 24% lower chance of being in the solid profile compared to the moderate profile.

#### Effects of the dichotomous covariates on the transition probabilities

Regarding the transition probabilities between the different profiles, there are consistent negative intercepts, which can be regarded as base logits if the dichotomous variables are equal to zero. Negative intercepts indicate low transition probabilities. Due to low logit values for the covariates (gender, immigration background and educational track) and the high standard errors, the influence of the covariates on the transition probabilities is insignificant and these are not reported here. However, as presented below, in the case where all covariates are set to zero, some transitions reach probabilities between 8% and 19%.

The transition probabilities from profiles 1 (‘low’), 3 (‘high’) and 4 (‘solid’) to Profile 2 (‘moderate’) are insignificant when controlling for the dichotomous covariates. Regarding the transition from Profile 2 (‘moderate’) to Profile 1 (‘low’), the transition probability is 17% and significant (Intercept = −1.61, *SE* = .29, 95% CI [−2.18, −1.04]) when all dichotomous covariates are set to zero. However, none of the covariates influence the transition probability significantly. Regarding the transition probability from Profile 1 (‘low’) to Profile 4 (‘solid’), there is a significant intercept (Intercept = −1.44, *SE* = .24, 95% CI [−1.91, −.97]) that can be interpreted as a probability of 19% when all covariates are set to zero. Finally, the transition probability from Profile 2 (‘moderate’) to Profile 3 (‘high’) is also significant (Intercept = −2.49, *SE* = .52, 95% CI [−3.51, −1.47]), when all covariates are set to zero. However, none of the covariates has a significant effect on the transition probability of 8%.

#### Effects of the dichotomous covariates on the initial probabilities and of the metric covariate on the transition probabilities (model 3)

The third model (LogLik = −15,849.70, AIC = 31,841.39, BIC = 32,210.94,) firstly displays the effects of the dichotomous covariates on the initial probabilities to be assigned to profiles ‘low’ (Profile 1), ‘solid’ (Profile 4) or ‘high’ (Profile 3) compared to the ‘moderate’ profile (Profile 2), when the effects of the dichotomous covariates on those probabilities are controlled. Secondly, it provides information about the effects of the students' levels of intrinsic motivation at t1 on the logit for the transition probabilities from profile to profile over time. Since the level of *intrinsic motivation* cannot assume the value 0, the intercept of the transition probabilities cannot be interpreted meaningfully in this model. Based on the intercept, it is therefore possible only to draw conclusions about the level of transition probability: Positive intercepts indicate higher transition probabilities, while negative intercepts indicate lower transition probabilities. The same applies to positive coefficients of the predictors (the students' levels of *intrinsic motivation*): Positive coefficients increase the transition probability, while negative coefficients decrease the transition probability to another profile. The log‐odd coefficients for the intercepts and the effects of the students' level of *intrinsic motivation*, their standard errors and 95% CI are displayed in Table 5.

**TABLE 5:** Log‐odds for the transition probabilities between the four profiles considering the students' intrinsic motivation (IM).

Logit = 1 (X1, ‘low’)						
	Moderate (X2)		High (X3)		Solid (X4)	
	*B* (*SE*)	95% CI	*B* (*SE*)	95% CI	*B* (*SE*)	95% CI
Intercept	**−2.38 (.41)**	**[−3.18, −1.58]**	−15.39 (2140.47)	[−4210.71, 4179.93]	12.23 (3678.40)	[−7197.43, 7221.89]
IM	.**28 (.13)**	**[.03, .53]**	−1.80 (2072.60)	[−4054.10, 4050.50]	−22.45 (3679.40)	[−7232.07, 7187.17]

Logit = 2 (X2, ‘moderate’)						
	Low (X1)		High (X3)		Solid (X4)	
	*B* (*SE*)	95% CI	*B* (*SE*)	95% CI	*B* (*SE*)	95% CI
Intercept	.99 (.84)	[−.66, 2.64]	**−4.13 (1.93)**	**[−7.93, −.35]**	.04 (1.00)	[−1.93, 2.01]
IM	−.24 (.27)	[−.77, .29]	.**89 (.45)**	**[.01, 1.77]**	−.23 (.32)	[−.86, .40]

Logit = 3 (X3, ‘high’)						
	Low (X1)		Moderate (X2)		Solid (X4)	
	*B* (*SE*)	95% CI	*B* (*SE*)	95% CI	*B* (*SE*)	95% CI
Intercept	−25.53 (23,252.12)	[−45,600.08, 45,548.62]	**−3.56 (.94)**	**[−5.40, −1.72]**	4.87 (16.18)	[−26.85, 36.60]
IM	1.07 (7277.12)	[−26.85, 36.60]	.23 (.25)	[−.26, .73]	−6.50 (15.67)	[−37.22, 24.21]

Logit = 4 (X4, ‘solid’)						
	Low (X1)		Moderate (X2)		High (X3)	
	*B* (*SE*)	95% CI	*B* (*SE*)	95% CI	*B* (*SE*)	95% CI
Intercept	**−1.50 (.36)**	**[−2.21, −.79]**	**−4.37 (1.20)**	**[−6.72, −2.02]**	−24.69 (7006.74)	[−12,757.89, 13,708.51]
IM	−.08 (.12)	[−.32, .15]	.10 (.38)	[−.65, .85]	1.12 (1941.34)	[−3799.80, 3802.05]

*Note*: Significant effects (p ≤ .05) are highlighted in bold.

### Transition probabilities to profile 1 (‘low’)

The transition probabilities from Profile 2 (‘moderate’) to Profile 1 (‘low’) show a negative intercept that can be interpreted as a low transition probability. Additionally, the logit for students' levels of *intrinsic motivation* has a positive effect on the transition probability from Profile 2 (‘moderate’) to Profile 1 (‘low’) (*B* = .28, *SE* = .13, 95% CI [.03, .53]). This coefficient can be interpreted as follows: When a student's level of intrinsic motivation increases by one unit, the transition probability increases by .28 units. Based on the Equation (1) stated earlier, the medium transition probability for a student within Profile 2 (‘moderate’) to Profile 1 (‘low’), with an *intrinsic motivation* value (*M* = 4.00) that exceeds the overall mean at t1 (*M* = 3.02), is calculated as follows:

$$p=\frac{e^{\text{logit}}}{1+e^{\text{logit}}}$$

$${\mathrm{Log}}_{4,1}=-2.38+\left(0.28\times 4\right)=-1.26$$

$$p_{4,1}=\frac{e^{-1.26}}{1+e^{-1.26}}=0.22$$

Thus, for students with an above‐average value for *intrinsic motivation* (*M* = 4.00), the probability of transitioning from Profile 2 to Profile 1 is 22%, while for students with below‐average values of intrinsic motivation (*M* = 1.00), the probability of transitioning is 10%.

### Transition probabilities to profile 2 (‘moderate’)

When interpreting the transition probabilities from Profile 3 (‘high’) to Profile 2 (‘moderate’), first, the negative intercept implies a low transition probability. Second, the analysis shows that students' levels of *intrinsic motivation* increase this transition probability. The transition probability increases by .89 units per one‐unit increase in students' levels of intrinsic motivation. The transition probability from the high profile to the moderate profile is 36% when students report higher levels of *intrinsic motivation* than the average (*M* = 4.00). On the other hand, when a student's level of *intrinsic motivation* is fixed at 1, the transition probability between these two profiles is 4% and thus much lower.

### Transition probabilities to profile 3 (‘high’)

For transitions to Profile 3 (‘high’), a significant intercept was found only for the transition from Profile 2 (‘moderate’) (*B* = −3.56, *SE* = .94, 95% CI [−5.40, −1.72]). The negative intercept hints at a low but significant transition probability from the moderate to the high profile. However, the probability cannot be interpreted further, since the level of intrinsic motivation cannot assume the value zero. The students' levels of intrinsic motivation also have no significant effect on the transition probability.

### Transition probabilities to profile 4 (‘solid’)

The negative intercepts regarding the transition probabilities from Profile 1 (‘low’) and Profile 2 (‘moderate’) to Profile 4 (‘solid’) are – based on the negative intercepts – rather low, but significant. However, no significant effects of the students' levels of intrinsic motivation on the transition probabilities were found.

## DISCUSSION

### Theoretical significance of the findings

This study aimed to explore the patterns and stability of early secondary school students' social relationships at school (with peers and teachers), focusing on how these relationships evolve within the first year of secondary school. By using latent profile and latent transition analysis, the main findings identify four relationship quality profiles with different stabilities over time, with students' intrinsic motivation being a relevant factor for the likelihood of moving towards better‐quality social relationships. The results can thus be interpreted in the light of previous research and our stated hypotheses as follows.

As assumed, and in line with Raufelder et al. (2013) and Burns et al. (2022), the latent profile analyses conducted in this study have shown that the four‐profile solution is the best fit for all time measurement points. The solution comprises one profile characterized by comparably low values, and one with high values on all measures of the quality of social relationships in class (H1a). We found – as anticipated in H1b – two other profiles, but we did not find a profile with a good quality teacher–student relationship and poor quality peer relationships, nor the other way around. Instead, we found an additional profile with comparably solid relationship quality values but with lower student focus on the teachers, and another profile with overall moderate values on all measures of the quality of social relationships in class.

Based on the findings of ANOVAs and Tukey tests, we have shown that the profiles differ significantly in terms of the students' levels of extrinsic and intrinsic motivation. Consistent with our expectations (H2a) and prior research results, students in the profile with more negative perceptions of the quality of their social relationships in class display significantly lower levels of intrinsic motivation compared to their peers in more favourable profiles (characterized by the perception of good social relationships), at each time measurement point (Burns et al., 2022; Kindermann, 1993; Roorda et al., 2011; Wentzel, 2017). Moreover, students who reported higher levels of extrinsic motivation are more likely to be assigned to unfavourable social relationship groups (low or moderate profiles), which is in line with our assumption that students who are extrinsically motivated might not be able to build up solid and high‐quality relationships.

The latent transition analyses show that most of the profiles are relatively stable, as assumed in H3 (see Figure 4). But the profiles that comprise the more positive perceptions of the quality of social relationships in class are the most stable, whereas the latent transition model without covariates reveals that the profile with the lowest values regarding the perceptions of the quality of social relationships displays the lowest stability. The findings show that students who were initially grouped in the profile with low scores change comparatively frequently to the profile with solid scores or even to a more positive assessment. This could indicate that students acclimate to the new school environment after an initial ‘shock’ (for example, they may initially lack a social network of peers because their friends from primary school transitioned to a different type of school). These students gradually build up a positive social network or begin to perceive their social environment more positively. But it may also be that the assessment of the quality of social relationships right at the beginning of the first year in secondary education may be confounded by the students' experiences in primary school. In other words, students who had negative experiences with regard to their social relationships in primary school may initially be assigned to the profile with low values, but due to positive experiences in secondary school could transit to more favourable profiles over the course of time.

Conversely, some students who were formerly assigned to the high profile switched to the low or moderate profiles. The explanation for this transition could be the opposite of the aforementioned declaration – namely that students who perceived a solid quality of social relationships in primary education may have a higher risk of having more negative experiences in secondary education and would thus transit to less favourable profiles. With the available data, we cannot clarify why these transitions take place in this way; follow‐up studies need to provide further information on the complex interconnectedness of these profiles, by, for example, investigating students' perceived social relationships from primary school onwards. But all in all, this result should be interpreted in a positive way, as it shows that belonging to the ‘low’ profile at the beginning of secondary school is not irreversible. Nevertheless, the results over time should be interpreted with caution, especially considering the low proportion of students in the low profile in this study.

Our further findings on the latent transition model with dichotomous covariates show that students' gender, cultural origin and educational track influence the initial probability of being assigned to one of the four profiles over time, as expected (H4a), and in line with prior research findings (Hughes & Cao, 2018; Spilt et al., 2012). We found that male students are less likely to be initially assigned to the profiles characterized by high or moderate quality of social relationships in class, compared to their initial probability of being assigned to the profile with the lowest values on all measures of quality. Furthermore, students who attend the higher educational track have a higher probability of being assigned to the profile with overall moderate values on all measures of the quality of social relationships in class, compared to being assigned to the profile with the lowest values on all those measures.

But, contrary to our assumptions, none of the transition probabilities are significantly influenced by the dichotomous covariates. Thus, it must be assumed that despite the differences regarding the initial probabilities, there are no more or less frequent transitions that could be traced back to students' gender, immigration background or their educational track. Further individual factors (achievement emotions, well‐being, need for additional support, etc.) and potential aspects of the learning environment (e.g., feedback quality, teacher engagement, implementation of a relationship support program) need to be investigated in order to understand more deeply the dynamics of students' social relationships after transitioning to secondary school.

Finally, our results show that the transition probability from Profile 3 (‘high’) to Profile 2 (‘moderate’) is increased by higher levels of intrinsic motivation. Thus, students with higher intrinsic motivation are more likely to transit to a profile with a less favourable perception of the quality of social relationships in class, which is in line with our assumption H4b. But the latent transition model with the students' levels of intrinsic motivation as a covariate affecting the transition probabilities shows that the low probability of transitioning from Profile 2 (‘moderate’) to Profile 1 (‘low’) increases with increasing levels of students' intrinsic motivation. Students in the profile characterized by moderate values who experience an increase in intrinsic motivation (e.g., through increased subject interest) might have higher expectations of lessons and social relationships at school, and attach more value to these (Wigfield & Eccles, 2000). If these expectations are not met, for example, through a higher quality of social relationships, then there is a higher risk of them transitioning to the most negative profile.

In sum, our findings underline the importance of fostering positive social relationships with both peers and teachers in school contexts, as such relationships appear to be very closely linked to student motivation. In particular, in this study, students in the ‘high’ profile exhibited the highest levels of intrinsic motivation, coupled with the lowest levels of extrinsic motivation. This pattern suggests that positive social relationships may help to create an environment where students are more self‐driven in their learning. In contrast, students in the ‘low’ profile demonstrated the inverse pattern, with low intrinsic motivation and high extrinsic motivation, highlighting the potentially detrimental impact of weaker social relationships on students' levels of internal motivation. An exception to this trend was observed at t3, where extrinsic motivation was higher in the ‘moderate’ group than in those with the 'low' profile. This nuance suggests that additional factors – such as external pressures or challenges of the post‐transition process, might temporarily influence student motivation. Overall, these results underscore the critical role of nurturing positive, supportive social relationships in the classroom, in order to also promote students' levels of intrinsic motivation.

### Strengths and limitations of the study

Our study has numerous strengths, but also some limitations. One of the most important strengths is that we conducted a longitudinal design study, with three time measurement points. We can therefore derive causal conclusions from our results. However, we analysed latent profile transitions only in the post‐transition phase (during the first year after transitioning from primary to secondary school); thus, we can refer only to the adjustment process after the transition, and not to the time immediately before or during the transition to the next type of school.

Additionally, we used only standardized and valid scales, showing good internal consistencies which merely displayed strong or even strict measurement invariance for groups as well as between the measurement time points (see Appendix S2 for measurement invariance). Another advantage is the comprehensive and large sample, which allowed us to also uncover small effects. Even so, we cannot rule out the possibility of biases regarding the effects of educational track and the students' gender (Markus et al., 2022), as the sample comprised a high percentage of female students, and no students from lower educational track schools. The proportion of girls in the sample from mono‐educational schools must also be addressed, as mono‐educational schools are controversial in terms of their effect on students' school well‐being (Obermeier & Gläser‐Zikuda, 2022; Schurt & Waburg, 2007).

The imbalance between the different social relationships (e.g., several aspects of teacher–student relationships and one aspect of student–student relationships) could have led to biases in formulating and analysing the profiles. Furthermore, the quality of peer relationships was measured using five recoded items depicting feelings of social relatedness (e.g., “Did it ever happen in the last few weeks that you felt like an outsider in your class?”); the absence of problems with the whole class (e.g., “Did it ever happen in the last few weeks that you had any problems with your class?”); or problems with single students (e.g., “Did it ever happen in the last few weeks that you had a problem with a friend at school?”). Since we recoded the answers, we measured the absence of social conflicts rather than positive social relationships with the whole class or single students, but we did not measure severe negative aspects of peer relationships (such as bullying) (Gubbels et al., 2019; Schlesier, Vierbuchen, & Matzner, 2023; Stearns & Glennie, 2006). Additionally, one scale of teacher–student relationships (namely student orientation) also includes one student–student relationship item, but given that it is only a single item and does not dominate the overall construct, we cannot derive substantial conclusions from that scale regarding peer relationships in class. Regarding the teacher–student relationship, we did not capture negative aspects (for example, conflicts) (Raufelder et al., 2016; Roeser & Eccles, 1998), as such negative relationship aspects might even push students out of school. Moreover, there is a clear predominance of variables capturing instructional practices, which are undoubtedly a central component of teacher–student relationships in school contexts (e. g., when perceived as supportive, clear, predictable, etc.), but they do not fully reflect the socio‐emotional core of these relationships. Therefore, future studies should continue to focus on the socio‐emotional dimensions of teacher–student relationships in order to develop a more comprehensive understanding of how these relationships evolve over the course of a school year. We also did not ask about specific school subjects, nor about the assessment of individual dyadic teacher–student relationships. The investigation of dyadic or triadic relationships and their links to students' motivation is certainly a promising avenue for future research, as it may offer deeper insights into the relevance of specific interpersonal connections. However, since the aim of the present study was to capture students' more global need for social relatedness and their overarching perception of school as a social context, we chose a broader assessment of social relationships within the school environment.

Nevertheless, our results appear to be highly relevant, confirming Jiang and Yang's (2022) finding that positive relationships are more closely related to student motivation. However, it can be assumed that specific questions about positive aspects of social relationships with peers (e.g., emotional support) or about serious social problems (such as bullying by peers) or even the inclusion of negative components about the teacher–student relationship (e.g., preference, disadvantage, conflict) might have shown different correlations. Therefore, in follow‐up studies, the findings should be replicated, with attention being paid to a more balanced inclusion of positive and negative dimensions of social relationships.

One other limitation concerns the operationalization of motivation. While Self‐Determination Theory (SDT) differentiates between various forms of motivation (e.g., external, introjected, identified and integrated), our analyses relied on a more global measure of motivation. Although a more fine‐grained approach might have yielded additional insights, the complexity of the present study (e. g., spanning multiple time points, relationship dimensions and motivational outcomes) required a certain level of parsimony to ensure conceptual clarity and interpretability. Future research could expand on these findings by incorporating more differentiated motivational constructs in longitudinal designs. We would also like to highlight the fact that while we examined extrinsic motivation in terms of its differences between the social relationship profiles, we did not include it in the LTA model. This decision was made for two reasons: first, the effects of extrinsic and intrinsic motivation would likely counteract each other within the LTA model; and second, the differences in extrinsic motivation between most profiles were not significant – whereas the differences in intrinsic motivation were substantial across profiles.

Another methodological aspect that should be discussed is the small size (<5%) of the ‘low’ profile. Although it has been found that such small profiles can be erroneous (Ferguson et al., 2020), we opted for this solution on the basis of theoretical and methodological assumptions, as follows. Firstly, this profile includes students who perceive all dimensions of the teacher–student relationship quite negatively and also maintain problems in their relationships with peers. The number of such students can theoretically be considered to be small. Since the theoretical interpretability of a profile (alongside statistical fit indices) is an important criterion for determining the appropriate number of profiles (Ferguson et al., 2020; Marsh et al., 2009), we decided to retain the small profile. Furthermore, the profile contains more than 30 students at each measurement point, which, according to Vincent and Weir (2012), is sufficient to draw initial conclusions. Nevertheless, such conclusions should be drawn cautiously.

The longitudinal design in this study offers great advantages in terms of generating causal findings and observing individual development, but it is also linked to challenges such as the change of teachers or changes in class composition (students who move away or move into the class; or students who leave for another educational track, or come from another educational track). Although it is common for the teaching staff in most German secondary schools to change every two years, the students in this study were taught by the same teachers at all three measurement points (this was confirmed by the participating schools). Nevertheless, there may have been a change of individual teachers in individual cases, which we were unfortunately unable to check. Another challenge with longitudinal data is the possibility of students dropping out from the study across the waves (see Table 1). However, in this study, the missing data points were completely missing at random for t1 (MCAR: *χ* ^2^(74) = 82.24, *p* = .24) and t2 (MCAR: *χ* ^2^(86) = 92.72, *p* = .29). At t3, only female students showed a significantly higher likelihood of missing data across the variables of interest. This increased probability may be attributable to the higher proportion of female participants in the sample (over 80%). We replaced missing data through multiple imputations using the random forest algorithm. Last but not least, the hierarchical structure of the data (students nested within schools) was not taken into account due to low ICC values (≤.05; LeBreton & Senter, 2008) for nearly all constructs related to perceived social relationships in class, with the exception of classroom management. However, most importantly, we applied a person‐centred approach based on the students' perceptions of the quality of social relationships in class, and thus contributed to the scarce findings that employ such approaches.

## CONCLUSION

All in all, we were able to conduct a comprehensive study which showed that four profiles can be distinguished in terms of the perceived quality of social relationships in class. The profiles vary in their stability, with those characterized by a low quality of social relationships being the least stable. As the relationship profiles are directly linked to student motivation, and profiles with the highest relationship quality reflect significantly higher levels of intrinsic motivation, it is clear that social relationships between students and their classmates and teachers should be strengthened. Based on the present study, we therefore recommend implementing programmes at schools that focus on improving relationships and instructional quality, so that students are more motivated overall, but particularly after transitioning to secondary school. Further studies should also integrate negative aspects of relationships in order to gain insights into the importance of both positive and negative social relationships in class.

## AUTHOR CONTRIBUTIONS

**Juliane Schlesier:** Conceptualization; formal analysis; investigation; methodology; visualization; writing – original draft; writing – review and editing; validation. **Ramona Obermeier:** Conceptualization; data curation; formal analysis; investigation; methodology; project administration; visualization; writing – original draft; writing – review and editing. **Michaela Gläser‐Zikuda:** Data curation; funding acquisition; investigation; project administration; writing – review and editing.

## CONFLICT OF INTEREST STATEMENT

The authors declare that they have no conflicts of interest.

## Supporting information


Appendix S1.–S5.

## Data Availability

The data that support the findings of this study are available from the corresponding author upon reasonable request.
