# Supplementary material for: Students' relationship quality in class: Exploring latent profiles, latent transitions and links to student motivation
Source: Br J Educ Psychol. 2025 Sep 10;95(4):1234–65. doi: 10.1111/bjep.70028 (PMC12590938; doi:10.1111/bjep.70028)
Supplement: Supplementary file 1 — Appendix S1.–S5. [file BJEP-95-1234-s001.zip › bjep70028-sup-0001-AppendixS1.rtf]

Appendix S1

Table A1 

Correlations with Confidence Intervals (t1)
 
Variable	1	2	3	4	5	6	7	8	9	
										
1. Ed. Track1	 	 	 	 	 	 	 	 	 	
 	 	 	 	 	 	 	 	 	 	
2. Gender1 	.18**	 	 	 	 	 	 	 	 	
 	[.12, .24]	 	 	 	 	 	 	 	 	
3. IB1	.05	.01	 	 	 	 	 	 	 	
 	[-.01, .11]	[-.05, .07]	 	 	 	 	 	 	 	
4. EM	-.07*	.13**	-.03	 	 	 	 	 	 	
 	[-.13, -.01]	[.08, .19]	[-.08, .03]	 	 	 	 	 	 	
5. IM	.11**	-.01	-.05	-.14**	 	 	 	 	 	
 	[.05, .17]	[-.07, .05]	[-.10, .01]	[-.19, -.08]	 	 	 	 	 	
6. SO	.09**	-.13**	.05	-.16**	.32**	 	 	 	 	
 	[.02, .15]	[-.19, -.07]	[-.01, .12]	[-.22, -.10]	[.27, .38]	 	 	 	 	
7. CM	.15**	-.11**	.07*	-.16**	.32**	.67**	 	 	 	
 	[.09, .21]	[-.17, -.05]	[.01, .13]	[-.22, -.10]	[.26, .37]	[.63, .70]	 	 	 	
8. CS	.10**	-.02	.04	-.13**	.43**	.67**	.57**	 	 	
 	[.04, .16]	[-.08, .05]	[-.02, .10]	[-.19, -.07]	[.38, .48]	[.64, .71]	[.52, .61]	 	 	
9. ACT	.07*	-.02	.04	-.11**	.20**	.27**	.29**	.22**	 	
 	[.01, .14]	[-.08, .05]	[-.03, .10]	[-.17, -.04]	[.13, .26]	[.21, .34]	[.22, .35]	[.15, .29]	 	
10. NSP	.08**	-.07*	.09**	-.25**	.07*	.36**	.39**	.28**	.12**	
 	[.02, .14]	[-.13, -.01]	[.03, .15]	[-.30, -.19]	[.01, .13]	[.30, .41]	[.33, .44]	[.22, .34]	[.05, .19]	
Note. * indicates p < .05. ** indicates p < .01. 1 Kendalls' Tau was calculated for correlations between dichotomous and metric variables, IB = Immigration Background, EM = Extrinsic Motivation, IM = Intrinsic Motivation, SO = Student Orientation of the Teachers, CM = Classroom Management, CS = Clarity and Structure, ACT = Activation During the Lessons, NSP = Absence of Social Problems with Peers
Table A2 
 
Correlations with Confidence Intervals (t2)
 
Variable	1	2	3	4	5	6	7	8	9	
										
1. Ed. Track1	 	 	 	 	 	 	 	 	 	
 	 	 	 	 	 	 	 	 	 	
2. Gender1 	.18**	 	 	 	 	 	 	 	 	
 	[.12, .24]	 	 	 	 	 	 	 	 	
3. IB1	.05	.01	 	 	 	 	 	 	 	
 	[-.01, .11]	[-.05, .07]	 	 	 	 	 	 	 	
4. EM	-.06*	.10**	-.04	 	 	 	 	 	 	
 	[-.12, -.00]	[.04, .16]	[-.10, .02]	 	 	 	 	 	 	
5. IM	.03	-.05	-.01	-.25**	 	 	 	 	 	
 	[-.03, .09]	[-.11, .01]	[-.07, .05]	[-.31, -.20]	 	 	 	 	 	
6. SO	.05	-.11**	.07*	-.24**	.33**	 	 	 	 	
 	[-.02, .11]	[-.18, -.05]	[.01, .13]	[-.29, -.18]	[.27, .38]	 	 	 	 	
7. CM	.10**	-.02	.08*	-.22**	.36**	.65**	 	 	 	
 	[.04, .16]	[-.08, .04]	[.02, .14]	[-.27, -.16]	[.30, .41]	[.62, .69]	 	 	 	
8. CS	.08*	-.07*	.02	-.24**	.39**	.67**	.61**	 	 	
 	[.02, .14]	[-.13, -.00]	[-.05, .08]	[-.30, -.19]	[.34, .44]	[.63, .70]	[.58, .65]	 	 	
9. ACT	.11**	-.01	.10**	-.13**	.38**	.51**	.48**	.55**	 	
 	[.05, .17]	[-.08, .05]	[.03, .16]	[-.18, -.07]	[.33, .43]	[.47, .56]	[.43, .52]	[.51, .59]	 	
10. NSP	.01	.01	.05	-.22**	.07*	.34**	.27**	.30**	.11**	
 	[-.06, .07]	[-.05, .07]	[-.01, .11]	[-.28, -.17]	[.01, .12]	[.29, .39]	[.22, .33]	[.25, .35]	[.05, .16]	
Note. 	* indicates p < .05. ** indicates p < .01. 1 Kendalls' Tau was calculated for correlations between dichotomous and metric variables, IB = Immigration Background, EM = Extrinsic Motivation, IM = Intrinsic Motivation, SO = Student Orientation of the Teachers, CM = Classroom Management, CS = Clarity and Structure, ACT = Activation During the Lessons, NSP = Absence of Social Problems with Peers
Table A3
 
Correlations with Confidence Intervals (t3)
 
Variable	1	2	3	4	5	6	7	8	9	
										
1. Ed. Track1	 	 	 	 	 	 	 	 	 	
 	 	 	 	 	 	 	 	 	 	
2. Gender1 	.18**	 	 	 	 	 	 	 	 	
 	[.12, .24]	 	 	 	 	 	 	 	 	
3. IB1	.05	.01	 	 	 	 	 	 	 	
 	[-.01, .11]	[-.05, .07]	 	 	 	 	 	 	 	
4. EM	-.04	.04	-.02	 	 	 	 	 	 	
 	[-.11, .02]	[-.02, .11]	[-.08, .05]	 	 	 	 	 	 	
5. IM	.03	-.06	-.01	-.23**	 	 	 	 	 	
 	[-.04, .09]	[-.12, .01]	[-.07, .06]	[-.28, -.17]	 	 	 	 	 	
6. SO	.03	-.09**	.06	-.21**	.34**	 	 	 	 	
 	[-.04, .10]	[-.16, -.03]	[-.00, .13]	[-.27, -.15]	[.28, .39]	 	 	 	 	
7. CM	.14**	-.03	.03	-.20**	.29**	.64**	 	 	 	
 	[.07, .20]	[-.09, .04]	[-.04, .10]	[-.25, -.14]	[.23, .35]	[.60, .67]	 	 	 	
8. CS	.09*	-.02	.03	-.22**	.37**	.69**	.61**	 	 	
 	[.02, .15]	[-.09, .04]	[-.04, .10]	[-.28, -.16]	[.31, .42]	[.66, .72]	[.57, .65]	 	 	
9. ACT	.08*	-.01	.03	-.15**	.37**	.47**	.45**	.54**	 	
 	[.01, .15]	[-.08, .06]	[-.04, .10]	[-.21, -.09]	[.31, .42]	[.42, .51]	[.40, .50]	[.50, .59]	 	
10. NSP	.08*	-.01	.05	-.25**	.10**	.35**	.31**	.35**	.17**	
 	[.02, .15]	[-.08, .06]	[-.01, .12]	[-.30, -.19]	[.04, .17]	[.30, .40]	[.26, .37]	[.29, .40]	[.11, .23]	
Note. 	* indicates p < .05. ** indicates p < .01. 1 Kendalls' Tau was calculated for correlations between dichotomous and metric variables, IB = Immigration Background, EM = Extrinsic Motivation, IM = Intrinsic Motivation, SO = Student Orientation of the Teachers, CM = Classroom Management, CS = Clarity and Structure, ACT = Activation During the Lessons, NSP = Absence of Social Problems with Peers
