# Supplementary material for: Students' relationship quality in class: Exploring latent profiles, latent transitions and links to student motivation
Source: Br J Educ Psychol. 2025 Sep 10;95(4):1234–65. doi: 10.1111/bjep.70028 (PMC12590938; doi:10.1111/bjep.70028)
Supplement: Supplementary file 1 — Appendix S1.–S5. [file BJEP-95-1234-s001.zip › bjep70028-sup-0002-AppendixS2.docx]

**Appendix S2**

**Table B1**

*Measurement Invariance Test of the Dimensions of Perceived Quality of Social Relationships*

| Invariance level | Model–Fit | | | | | | | |  | | χ^2^–difference test | | | |
| --- | --- | --- | --- | --- | --- | --- | --- | --- | --- | --- | --- | --- | --- | --- |
| Student orientation |  | | | | | | | |  | |  | | | |
|  | χ^2^ | df | | *p* | | CFI | | RMSEA |  | | ∆ χ^2^ | | ∆ df | *p* |
| Configural | 288.70 | 114 | | .000 | | .997 | | .034 |  | |  | |  |  |
| Metric | 295.02 | 122 | | .000 | | .997 | | .033 |  | | 7.31 | | 8 | .503 |
| Scalar | 341.54 | 156 | | .000 | | .997 | | .030 |  | | 72.51 | | 34 | .000 |
| Strict | 372.24 | 168 | | .000 | | .996 | | .030 |  | | 29.30 | | 12 | .003 |
| Classroom management | | | | | | | | | | | | | | |
|  | χ^2^ | df | | *p* | | CFI | | RMSEA |  | | ∆ χ^2^ | | ∆ df | *p* |
| Configural | 275.62 | 72 | | .000 | | .987 | | .046 |  | |  | |  |  |
| Metric | 293.93 | 78 | | .000 | | .986 | | .046 |  | | 18.58 | | 6 | .005 |
| Scalar | 332,64 | 106 | | .000 | | .985 | | .040 |  | | 55.75 | | 28 | .001 |
| Strict | 389.29 | 116 | | .000 | | .982 | | .042 |  | | 64.28 | | 10 | .000 |
| clarity and structure | | | | | | | | |  | |  | | | |
|  | χ^2^ | df | | *p* | | CFI | | RMSEA |  | | ∆ χ^2^ | | ∆ df | *p* |
| Configural | 63.94 | 72 | | .740 | | 1.00 | | .000 |  | |  | |  |  |
| Metric | 79.95 | 78 | | .417 | | 1.00 | | .004 |  | | 18.70 | | 6 | .005 |
| Scalar | 105.87 | 106 | | .485 | | 1.00 | | .000 |  | | 41.48 | | 28 | .049 |
| Strict | 136.37 | 116 | | .095 | | .999 | | .012 |  | | 34.49 | | 10 | .000 |
| Absence of social problems | | | | | | | | | | | | | | |
|  | χ^2^ | | df | | *p* | | CFI | RMSEA | |  | | ∆ χ^2^ | ∆ df | *p* |
| Configural | 170.55 | | 72 | | .000 | | .998 | .032 | |  | |  |  |  |
| Metric | 174.07 | | 78 | | .000 | | .998 | .030 | |  | | 6.40 | 6 | .380 |
| Scalar | 520.77 | | 106 | | .000 | | .990 | .054 | |  | | 334.57 | 28 | .000 |
| Strict | 597.44 | | 116 | | .000 | | .988 | .056 | |  | | 86.98 | 10 | .000 |
| Activation | | | | | | | | | | | | | | |
|  | χ^2^ | | df | | *p* | | CFI | RMSEA | |  | | ∆ χ^2^ | ∆ df | *p* |
| Configural | 293.07 | | 114 | | .000 | | .990 | .035 | |  | |  |  |  |
| Metric | 296.49 | | 122 | | .000 | | .990 | .033 | |  | | 3.22 | 8 | .920 |
| Scalar | 370.89 | | 156 | | .000 | | .988 | .032 | |  | | 83.29 | 34 | .000 |
| Strict | 382.71 | | 168 | | .000 | | .988 | .031 | |  | | 13.47 | 12 | .336 |

**Table B2**

*Measurement Invariance Test of the Dimensions of Perceived Quality of Social Relationships and Students’ Motivation for Males and Females*

| Invariance level | Model–Fit | | | | | | | |  | |  | | | |
| --- | --- | --- | --- | --- | --- | --- | --- | --- | --- | --- | --- | --- | --- | --- |
| Extrinsic motivation t1 | | | | | | | | |  | |  | | | |
|  | χ^2^ | df | | *p* | | CFI | | RMSEA |  | | ∆ CFI | | ∆ RMSEA | |
| Configural | 30.575 | 10 | | .001 | | .983 | | .061 |  | |  | |  |  |
| Metric | 33.638 | 14 | | .002 | | .984 | | .050 |  | | + .001 | | - .011 |  |
| Scalar | 34.693 | 18 | | .010 | | .986 | | .042 |  | | + .002 | | - .008 |  |
| Strict | 49.846 | 23 | | .001 | | .978 | | .047 |  | | - .008 | | + .005 |  |
| Extrinsic motivation t2 | | | | | | | | | | | | | | |
|  | χ^2^ | df | | *p* | | CFI | | RMSEA |  | | ∆ CFI | | ∆ RMSEA | |
| Configural | 51.457 | 10 | | .000 | | .967 | | .090 |  | |  | |  |  |
| Metric | 54.632 | 14 | | .000 | | .968 | | .076 |  | | + .001 | | - .014 |  |
| Scalar | 60.999 | 18 | | .000 | | .965 | | .069 |  | | - .003 | | - .007 |  |
| Strict | 74.308 | 23 | | .000 | | .958 | | .067 |  | | - .007 | | - .002 |  |
| Extrinsic motivation t3 | | | | | | | | |  | |  | | | |
|  | χ^2^ | df | | *p* | | CFI | | RMSEA |  | | ∆ CFI | | ∆ RMSEA | |
| Configural | 60.478 | 10 | | .000 | | .946 | | .109 |  | |  | |  |  |
| Metric | 70.071 | 14 | | .000 | | .940 | | .097 |  | | - .006 | | - .012 |  |
| Scalar | 74.168 | 18 | | .000 | | .940 | | .087 |  | | --- | | - .010 |  |
| Strict | 84.498 | 23 | | .000 | | .934 | | .080 |  | | - .006 | | - .007 |  |
| Intrinsic motivation t1 | | | | | | | | | | | | | | |
|  | χ^2^ | | df | | *p* | | CFI | RMSEA | |  | | ∆ CFI | ∆ RMSEA | |
| Configural | 0.000 | | 00 | | .000 | | 1.000 | .000 | |  | |  |  |  |
| Metric | 8.212 | | 2 | | .016 | | .996 | .076 | |  | | - .004 | + .076 |  |
| Scalar | 9.112 | | 4 | | .058 | | .997 | .049 | |  | | + .001 | - .025 |  |
| Strict | 10.299 | | 7 | | .172 | | .997 | .030 | |  | | --- | - .019 |  |
| Intrinsic motivation t2 | | | | | | | | | | | | | | |
|  | χ^2^ | | df | | *p* | | CFI | RMSEA | |  | | ∆ CFI | ∆ RMSEA | |
| Configural | 0.000 | | 00 | | .000 | | 1.000 | .000 | |  | |  |  |  |
| Metric | 6.447 | | 2 | | .040 | | .997 | .067 | |  | | - .003 | + .067 |  |
| Scalar | 6.711 | | 4 | | .152 | | .998 | .037 | |  | | + .001 | - .030 |  |
| Strict | 17.354 | | 7 | | .015 | | .994 | .055 | |  | | --- | + .017 |  |
| Intrinsic motivation t3 | | | | | | | | | | | | | | |
|  | χ^2^ | | df | | *p* | | CFI | RMSEA | |  | | ∆ CFI | ∆ RMSEA | |
| Configural | 0.000 | | 00 | | .000 | | 1.000 | .000 | |  | |  |  |  |
| Metric | 0.863 | | 2 | | .650 | | 1.000 | .000 | |  | | --- | --- |  |
| Scalar | 5.920 | | 4 | | .205 | | .999 | .034 | |  | | - .001 | .034 |  |
| Strict | 9.827 | | 7 | | .199 | | .998 | .031 | |  | | - .001 | - .003 |  |
| Absence of social problems t1 | | | | | | | | | | | | | | |
|  | χ^2^ | | df | | *p* | | CFI | RMSEA | |  | | ∆ CFI | ∆ RMSEA | |
| Configural | 59.555 | | 10 | | .000 | | ,972 | .099 | |  | |  |  |  |
| Metric | 67.793 | | 14 | | .000 | | .970 | .087 | |  | | - .002 | - .012 |  |
| Scalar | 84.800 | | 18 | | .000 | | .962 | .087 | |  | | - .008 | --- |  |
| Strict | 92.857 | | 23 | | .000 | | .960 | .079 | |  | | - .002 | - .008 |  |
| Absence of social problems t2 | | | | | | | | | | | | | | |
|  | χ^2^ | | df | | *p* | | CFI | RMSEA | |  | | ∆ CFI | ∆ RMSEA | |
| Configural | 95.978 | | 10 | | .000 | | .960 | .132 | |  | |  |  |  |
| Metric | 108.205 | | 14 | | .000 | | .956 | .117 | |  | | - .004 | - .015 |  |
| Scalar | 116.272 | | 18 | | .000 | | .953 | .107 | |  | | - .003 | - .010 |  |
| Strict | 125.591 | | 23 | | .000 | | .951 | .096 | |  | | - .002 | - .011 |  |
| Absence of social problems t3 | | | | | | | | | | | | | | |
|  | χ^2^ | | df | | *p* | | CFI | RMSEA | |  | | ∆ CFI | ∆ RMSEA | |
| Configural | 129.088 | | 10 | | .000 | | .934 | .170 | |  | |  |  |  |
| Metric | 130.689 | | 14 | | .000 | | .935 | .142 | |  | | + .001 | - .018 |  |
| Scalar | 136.112 | | 18 | | .000 | | .935 | .127 | |  | | --- | - .015 |  |
| Strict | 153.755 | | 23 | | .000 | | .928 | .119 | |  | | - .007 | - .008 |  |
| Classroom management t1 | | | | | | | | | | | | | | |
|  | χ^2^ | | df | | *p* | | CFI | RMSEA | |  | | ∆ CFI | ∆ RMSEA | |
| Configural | 59.788 | | 10 | | .000 | | .945 | .100 | |  | |  |  |  |
| Metric | 72.879 | | 14 | | .000 | | .934 | .092 | |  | | - .011 | - .008 |  |
| Scalar | 122.001 | | 18 | | .000 | | .884 | .110 | |  | | - .050 | + .018 |  |
| Strict | 139.874 | | 23 | | .000 | | .869 | .103 | |  | | - .015 | - .007 |  |
| Classroom management t2 | | | | | | | | | | | | | | |
|  | χ^2^ | | df | | *p* | | CFI | RMSEA | |  | | ∆ CFI | ∆ RMSEA | |
| Configural | 83.904 | | 10 | | .000 | | .917 | .123 | |  | |  |  |  |
| Metric | 85.469 | | 14 | | .000 | | .920 | .103 | |  | | + .003 | - .020 |  |
| Scalar | 94.007 | | 18 | | .000 | | .914 | .095 | |  | | - .006 | - .008 |  |
| Strict | 102.049 | | 23 | | .000 | | .910 | .086 | |  | | - .004 | - .009 |  |
| Classroom management t3 | | | | | | | | | | | | | | |
|  | χ^2^ | | df | | *p* | | CFI | RMSEA | |  | | ∆ CFI | ∆ RMSEA | |
| Configural | 141.936 | | 10 | | .000 | | .894 | .170 | |  | |  |  |  |
| Metric | 138.986 | | 14 | | .000 | | .892 | .146 | |  | | - .002 | - .024 |  |
| Scalar | 145.837 | | 18 | | .000 | | .888 | .132 | |  | | - .004 | - .014 |  |
| Strict | 160.591 | | 23 | | .000 | | .879 | .121 | |  | | - .009 | - .011 |  |
| Clarity and structure t1 | | | | | | | | | | | | | | |
|  | χ^2^ | | df | | *p* | | CFI | RMSEA | |  | | ∆ CFI | ∆ RMSEA | |
| Configural | 24.148 | | 10 | | .007 | | .992 | .055 | |  | |  |  |  |
| Metric | 27.638 | | 14 | | .016 | | .992 | .046 | |  | | --- | - .009 |  |
| Scalar | 32.280 | | 18 | | .020 | | .992 | .042 | |  | | --- | - .004 |  |
| Strict | 45.876 | | 23 | | .003 | | .987 | .047 | |  | | - .005 | + .005 |  |
| Clarity and structure t2 | | | | | | | | | | | | | | |
|  | χ^2^ | | df | | *p* | | CFI | RMSEA | |  | | ∆ CFI | ∆ RMSEA | |
| Configural | 22.025 | | 10 | | .015 | | .994 | .051 | |  | |  |  |  |
| Metric | 29.695 | | 14 | | .008 | | .992 | .049 | |  | | - .002 | - .002 |  |
| Scalar | 34.927 | | 18 | | .010 | | .991 | .046 | |  | | - .001 | - .003 |  |
| Strict | 42.186 | | 23 | | .009 | | .990 | .043 | |  | | - .001 | - .003 |  |
| Clarity and structure t3 | | | | | | | | | | | | | | |
|  | χ^2^ | | df | | *p* | | CFI | RMSEA | |  | | ∆ CFI | ∆ RMSEA | |
| Configural | 32.097 | | 10 | | .000 | | .987 | .073 | |  | |  |  |  |
| Metric | 32.773 | | 14 | | .003 | | .989 | .057 | |  | | + .002 | - .015 |  |
| Scalar | 40.178 | | 18 | | .002 | | .987 | .055 | |  | | - .002 | - .002 |  |
| Strict | 45.877 | | 23 | | .003 | | .987 | .050 | |  | | --- | - .005 |  |
| Activation t1 | | | | | | | | | | | | | | |
|  | χ^2^ | | df | | *p* | | CFI | RMSEA | |  | | ∆ CFI | ∆ RMSEA | |
| Configural | 52.014 | | 18 | | .000 | | .966 | .064 | |  | |  |  |  |
| Metric | 68.734 | | 23 | | .000 | | .954 | .065 | |  | | - .012 | + .001 |  |
| Scalar | 84.812 | | 28 | | .000 | | .940 | .068 | |  | | - .014 | + .003 |  |
| Strict | 92.529 | | 34 | | .000 | | .938 | .063 | |  | | - .002 | - .005 |  |
| Activation t2 | | | | | | | | | | | | | | |
|  | χ^2^ | | df | | *p* | | CFI | RMSEA | |  | | ∆ CFI | ∆ RMSEA | |
| Configural | 69.275 | | 18 | | .000 | | .952 | .078 | |  | |  |  |  |
| Metric | 86.650 | | 23 | | .000 | | .940 | .077 | |  | | - .012 | - .002 |  |
| Scalar | 86.587 | | 28 | | .000 | | .944 | .069 | |  | | + .004 | - .008 |  |
| Strict | 98.354 | | 34 | | .000 | | .939 | .066 | |  | | - .005 | - .003 |  |
| Activation t3 | | | | | | | | | | | | | | |
|  | χ^2^ | | df | | *p* | | CFI | RMSEA | |  | | ∆ CFI | ∆ RMSEA | |
| Configural | 57.570 | | 18 | | .000 | | .961 | .073 | |  | |  |  |  |
| Metric | 64.905 | | 23 | | .000 | | .959 | .066 | |  | | - .002 | - .007 |  |
| Scalar | 64.423 | | 28 | | .000 | | .963 | .058 | |  | | - .003 | - .008 |  |
| Strict | 67.795 | | 34 | | .001 | | .966 | .051 | |  | | + .003 | - .007 |  |
| Student orientation t1 | | | | | | | | | | | | | | |
|  | χ^2^ | | df | | *p* | | CFI | RMSEA | |  | | ∆ CFI | ∆ RMSEA | |
| Configural | 94.514 | | 18 | | .000 | | .966 | .094 | |  | |  |  |  |
| Metric | 101.090 | | 23 | | .000 | | .965 | .084 | |  | | - .001 | - .010 |  |
| Scalar | 124.740 | | 28 | | .000 | | .956 | .087 | |  | | + .001 | + .003 |  |
| Strict | 165.104 | | 34 | | .000 | | .941 | .092 | |  | | - .015 | + .005 |  |
| Student orientation t2 | | | | | | | | | | | | | | |
|  | χ^2^ | | df | | *p* | | CFI | RMSEA | |  | | ∆ CFI | ∆ RMSEA | |
| Configural | 109.620 | | 18 | | .000 | | .964 | .103 | |  | |  |  |  |
| Metric | 120.456 | | 23 | | .000 | | .962 | .094 | |  | | - .002 | - .009 |  |
| Scalar | 171.592 | | 28 | | .000 | | .942 | .106 | |  | | - .020 | + .016 |  |
| Strict | 186.938 | | 34 | | .000 | | .938 | .100 | |  | | - .005 | - .006 |  |
| Student orientation t3 | | | | | | | | | | | | | | |
|  | χ^2^ | | df | | *p* | | CFI | RMSEA | |  | | ∆ CFI | ∆ RMSEA | |
| Configural | 158.501 | | 18 | | .000 | | .942 | .137 | |  | |  |  |  |
| Metric | 161.995 | | 23 | | .000 | | .943 | .120 | |  | | + .001 | - .017 |  |
| Scalar | 193.323 | | 28 | | .000 | | .930 | .122 | |  | | - .013 | + .002 |  |
| Strict | 203.394 | | 34 | | .000 | | .928 | .112 | |  | | - .002 | - .010 |  |

**Table B3**

*Measurement Invariance Test of the Dimensions of Perceived Quality of Social Relationships and Students’ Motivation for Students with and without Immigration Background*

| Invariance level | Model–Fit | | | | | | | |  | |  | | | |
| --- | --- | --- | --- | --- | --- | --- | --- | --- | --- | --- | --- | --- | --- | --- |
| Extrinsic motivation t1 | | | | | | | | |  | |  | | | |
|  | χ^2^ | df | | *p* | | CFI | | RMSEA |  | | ∆ CFI | | ∆ RMSEA | |
| Configural | 29.394 | 10 | | .001 | | .985 | | .059 |  | |  | |  |  |
| Metric | 34.109 | 14 | | .002 | | .984 | | .051 |  | | - .001 | | - .008 |  |
| Scalar | 51.645 | 18 | | .000 | | .974 | | .059 |  | | - .010 | | + .008 |  |
| Strict | 55.795 | 23 | | .000 | | .974 | | .051 |  | | --- | | - .008 |  |
| Extrinsic motivation t2 | | | | | | | | | | | | | | |
|  | χ^2^ | df | | *p* | | CFI | | RMSEA |  | | ∆ CFI | | ∆ RMSEA | |
| Configural | 44.615 | 10 | | .000 | | .973 | | .082 |  | |  | |  |  |
| Metric | 48.810 | 14 | | .000 | | .973 | | .069 |  | | --- | | - .013 |  |
| Scalar | 48.921 | 18 | | .000 | | .975 | | .058 |  | | + .002 | | - .011 |  |
| Strict | 62.987 | 23 | | .000 | | .968 | | .059 |  | | - .007 | | + .001 |  |
| Extrinsic motivation t3 | | | | | | | | |  | |  | | | |
|  | χ^2^ | df | | *p* | | CFI | | RMSEA |  | | ∆ CFI | | ∆ RMSEA | |
| Configural | 57.580 | 10 | | .000 | | .949 | | .105 |  | |  | |  |  |
| Metric | 58.677 | 14 | | .000 | | .952 | | .086 |  | | + .003 | | - .019 |  |
| Scalar | 72.062 | 18 | | .000 | | .942 | | .084 |  | | - .010 | | - .002 |  |
| Strict | 74.471 | 23 | | .000 | | .945 | | .073 |  | | + .003 | | - .011 |  |
| Intrinsic motivation t1 | | | | | | | | | | | | | | |
|  | χ^2^ | | df | | *p* | | CFI | RMSEA | |  | | ∆ CFI | ∆ RMSEA | |
| Configural | 0.00 | | 00 | | --- | | 1.00 | .000 | |  | |  |  |  |
| Metric | 3.743 | | 2 | | .154 | | .999 | .040 | |  | | - .001 | + .040 |  |
| Scalar | 8.025 | | 4 | | .091 | | .998 | .043 | |  | | - .00 | + .003 |  |
| Strict | 15.378 | | 7 | | .031 | | .995 | .047 | |  | | - .003 | + .004 |  |
| Intrinsic motivation t2 | | | | | | | | | | | | | | |
|  | χ^2^ | | df | | *p* | | CFI | RMSEA | |  | | ∆ CFI | ∆ RMSEA | |
| Configural | 0.00 | | 00 | | --- | | 1.00 | .000 | |  | |  |  |  |
| Metric | 1.126 | | 2 | | .569 | | 1.00 | .000 | |  | | --- | --- |  |
| Scalar | 8.476 | | 4 | | .076 | | .997 | .047 | |  | | - .003 | + .047 |  |
| Strict | 9.432 | | 7 | | .223 | | .999 | .026 | |  | | + .02 | - .021 |  |
| Intrinsic motivation t3 | | | | | | | | | | | | | | |
|  | χ^2^ | | df | | *p* | | CFI | RMSEA | |  | | ∆ CFI | ∆ RMSEA | |
| Configural | 0.00 | | 00 | | --- | | 1.00 | .000 | |  | |  |  |  |
| Metric | 0.731 | | 2 | | .694 | | 1.00 | .000 | |  | | --- | --- |  |
| Scalar | 1.812 | | 4 | | .770 | | 1.00 | .000 | |  | | --- | --- |  |
| Strict | 4.700 | | 7 | | .696 | | 1.00 | .000 | |  | | --- | --- |  |
| Absence of social problems t1 | | | | | | | | | | | | | | |
|  | χ^2^ | | df | | *p* | | CFI | RMSEA | |  | | ∆ CFI | ∆ RMSEA | |
| Configural | 71.585 | | 10 | | .000 | | .965 | .109 | |  | |  |  |  |
| Metric | 74.503 | | 14 | | .000 | | .966 | .092 | |  | | + .001 | - .017 |  |
| Scalar | 84.861 | | 18 | | .000 | | .962 | .086 | |  | | - .004 | - .006 |  |
| Strict | 104.232 | | 23 | | .000 | | .954 | .084 | |  | | - .008 | - .002 |  |
| Absence of social problems t2 | | | | | | | | | | | | | | |
|  | χ^2^ | | df | | *p* | | CFI | RMSEA | |  | | ∆ CFI | ∆ RMSEA | |
| Configural | 118.335 | | 10 | | .000 | | .950 | .147 | |  | |  |  |  |
| Metric | 125.594 | | 14 | | .000 | | .949 | .126 | |  | | - .001 | - .021 |  |
| Scalar | 126.194 | | 18 | | .000 | | .949 | .111 | |  | | --- | - .011 |  |
| Strict | 132.749 | |  | |  | | .948 | .099 | |  | | - .001 | - .012 |  |
| Absence of social problems t3 | | | | | | | | | | | | | | |
|  | χ^2^ | | df | | *p* | | CFI | RMSEA | |  | | ∆ CFI | ∆ RMSEA | |
| Configural | 135.491 | | 10 | | .000 | | .931 | .173 | |  | |  |  |  |
| Metric | 140.436 | | 14 | | .000 | | .930 | .146 | |  | | - .001 | - .027 |  |
| Scalar | 142.619 | | 18 | | .000 | | .931 | .130 | |  | | + .001 | - .016 |  |
| Strict | 151.593 | | 23 | | .000 | | .929 | .116 | |  | | - .002 | - .014 |  |
| Classroom management t1 | | | | | | | | | | | | | | |
|  | χ^2^ | | df | | *p* | | CFI | RMSEA | |  | | ∆ CFI | ∆ RMSEA | |
| Configural | 50.086 | | 10 | | .000 | | .956 | .089 | |  | |  |  |  |
| Metric | 52.665 | | 14 | | .000 | | .957 | .074 | |  | | + .001 | - .015 |  |
| Scalar | 60.486 | | 18 | | .000 | | .953 | .070 | |  | | - .003 | - .004 |  |
| Strict | 69.753 | | 23 | | .000 | | .948 | .065 | |  | | - .005 | - .005 |  |
| Classroom management t2 | | | | | | | | | | | | | | |
|  | χ^2^ | | df | | *p* | | CFI | RMSEA | |  | | ∆ CFI | ∆ RMSEA | |
| Configural | 84.067 | | 10 | | .000 | | .918 | .123 | |  | |  |  |  |
| Metric | 87.441 | | 14 | | .000 | | .919 | .103 | |  | | + .001 | - .020 |  |
| Scalar | 98.917 | | 18 | | .000 | | .910 | .097 | |  | | - .009 | - .005 |  |
| Strict | 108.758 | | 23 | | .000 | | .904 | .088 | |  | | - .006 | - .009 |  |
| Classroom management t3 | | | | | | | | | | | | | | |
|  | χ^2^ | | df | | *p* | | CFI | RMSEA | |  | | ∆ CFI | ∆ RMSEA | |
| Configural | 113.731 | | 10 | | .000 | | .910 | .156 | |  | |  |  |  |
| Metric | 122.263 | | 14 | | .000 | | .907 | .134 | |  | | - .003 | - .022 |  |
| Scalar | 123.747 | | 18 | | .000 | | .908 | .118 | |  | | + .001 | - .016 |  |
| Strict | 140.998 | | 23 | | .000 | | .897 | .111 | |  | | - .011 | - .007 |  |
| Clarity and structure t1 | | | | | | | | | | | | | | |
|  | χ^2^ | | df | | *p* | | CFI | RMSEA | |  | | ∆ CFI | ∆ RMSEA | |
| Configural | 10.827 | | 10 | | .371 | | 1.00 | .013 | |  | |  |  |  |
| Metric | 14.343 | | 14 | | .424 | | 1.00 | .007 | |  | | --- | - .006 |  |
| Scalar | 21.836 | | 18 | | .239 | | .998 | .022 | |  | | - .002 | + .015 |  |
| Strict | 31.847 | | 23 | | .103 | | .995 | .029 | |  | | - .003 | + .007 |  |
| Clarity and structure t2 | | | | | | | | | | | | | | |
|  | χ^2^ | | df | | *p* | | CFI | RMSEA | |  | | ∆ CFI | ∆ RMSEA | |
| Configural | 22.536 | | 10 | | .013 | | .993 | .051 | |  | |  |  |  |
| Metric | 24.726 | | 14 | | .037 | | .994 | .040 | |  | | + .001 | - .011 |  |
| Scalar | 29.644 | | 18 | | .041 | | .994 | .038 | |  | | --- | - .002 |  |
| Strict | 32.706 | | 23 | | .086 | | .995 | .030 | |  | | + .001 | - .008 |  |
| Clarity and structure t3 | | | | | | | | | | | | | | |
|  | χ^2^ | | df | | *p* | | CFI | RMSEA | |  | | ∆ CFI | ∆ RMSEA | |
| Configural | 36.024 | | 10 | | .000 | | .985 | .079 | |  | |  |  |  |
| Metric | 37.334 | | 14 | | .001 | | .987 | .063 | |  | | + .003 | - .016 |  |
| Scalar | 38.062 | | 18 | | .004 | | .989 | .052 | |  | | + .002 | - .011 |  |
| Strict | 55.045 | | 23 | | .000 | | .982 | .058 | |  | | - .007 | + .006 |  |
| Activation t1 | | | | | | | | | | | | | | |
|  | χ^2^ | | df | | *p* | | CFI | RMSEA | |  | | ∆ CFI | ∆ RMSEA | |
| Configural | 49.785 | | 18 | | .000 | | .968 | .061 | |  | |  |  |  |
| Metric | 54.655 | | 23 | | .000 | | .969 | .054 | |  | | + .001 | - .007 |  |
| Scalar | 55.836 | | 28 | | .001 | | .971 | .047 | |  | | + .002 | - .007 |  |
| Strict | 70.556 | | 34 | | .000 | | .961 | .049 | |  | | - .010 | + .002 |  |
| Activation t2 | | | | | | | | | | | | | | |
|  | χ^2^ | | df | | *p* | | CFI | RMSEA | |  | | ∆ CFI | ∆ RMSEA | |
| Configural | 66.068 | | 18 | | .000 | | .955 | .075 | |  | |  |  |  |
| Metric | 72.572 | | 23 | | .000 | | .952 | .068 | |  | | - .003 | - .007 |  |
| Scalar | 67.241 | | 28 | | .000 | | .962 | .056 | |  | | + .010 | - .008 |  |
| Strict | 79.030 | | 34 | | .000 | | .957 | .055 | |  | | - .005 | - .001 |  |
| Activation t3 | | | | | | | | | | | | | | |
|  | χ^2^ | | df | | *p* | | CFI | RMSEA | |  | | ∆ CFI | ∆ RMSEA | |
| Configural | 49.232 | | 18 | | .000 | | .970 | .064 | |  | |  |  |  |
| Metric | 50.098 | | 23 | | .001 | | .974 | .053 | |  | | + .004 | - .011 |  |
| Scalar | 56.325 | | 28 | | .001 | | .972 | .051 | |  | | - .002 | - .002 |  |
| Strict | 63.599 | | 34 | | .002 | | .971 | .047 | |  | | - .001 | - .004 |  |
| Student orientation t1 | | | | | | | | | | | | | | |
|  | χ^2^ | | df | | *p* | | CFI | RMSEA | |  | | ∆ CFI | ∆ RMSEA | |
| Configural | 105.404 | | 18 | | .000 | | .963 | .099 | |  | |  |  |  |
| Metric | 110.820 | | 23 | | .000 | | .963 | .088 | |  | | --- | - .011 |  |
| Scalar | 108.397 | | 28 | | .000 | | .965 | .079 | |  | | + .002 | - .009 |  |
| Strict | 117.334 | | 34 | | .000 | | .964 | .073 | |  | | - .001 | - .006 |  |
| Student orientation t2 | | | | | | | | | | | | | | |
|  | χ^2^ | | df | | *p* | | CFI | RMSEA | |  | | ∆ CFI | ∆ RMSEA | |
| Configural | 112.443 | | 18 | | .000 | | .964 | .104 | |  | |  |  |  |
| Metric | 126.082 | | 23 | | .000 | | .961 | .096 | |  | | - .003 | - .008 |  |
| Scalar | 137.617 | | 28 | | .000 | | .957 | .092 | |  | | - .004 | - .004 |  |
| Strict | 144.241 | | 34 | | .000 | | .957 | .084 | |  | | --- | - .008 |  |
| Student orientation t3 | | | | | | | | | | | | | | |
|  | χ^2^ | | df | | *p* | | CFI | RMSEA | |  | | ∆ CFI | ∆ RMSEA | |
| Configural | 150.567 | | 18 | | .000 | | .946 | .132 | |  | |  |  |  |
| Metric | 160.075 | | 23 | | .000 | | .944 | .118 | |  | | - .002 | - .014 |  |
| Scalar | 165.462 | | 28 | | .000 | | .942 | .110 | |  | | - .002 | - .008 |  |
| Strict | 179.472 | | 34 | | .000 | | .939 | .103 | |  | | - .003 | - .007 |  |
