# Supplementary material for: Students' relationship quality in class: Exploring latent profiles, latent transitions and links to student motivation
Source: Br J Educ Psychol. 2025 Sep 10;95(4):1234–65. doi: 10.1111/bjep.70028 (PMC12590938; doi:10.1111/bjep.70028)
Supplement: Supplementary file 1 — Appendix S1.–S5. [file BJEP-95-1234-s001.zip › bjep70028-sup-0003-AppendixS3.docx]

**Appendix S3**

**Output C1**

*CFA and List of Items*

***Social relationships t1***

lavaan 0.6.17 ended normally after 109 iterations

Estimator ML

Optimization method NLMINB

Number of model parameters 91

Used Total

Number of observations 1053 1346

Number of missing patterns 102

Model Test User Model:

Test statistic 1167.460

Degrees of freedom 314

P-value (Chi-square) 0.000

Model Test Baseline Model:

Test statistic 10719.241

Degrees of freedom 351

P-value 0.000

User Model versus Baseline Model:

Comparative Fit Index (CFI) 0.918

Tucker-Lewis Index (TLI) 0.908

Robust Comparative Fit Index (CFI) 0.916

Robust Tucker-Lewis Index (TLI) 0.906

Loglikelihood and Information Criteria:

Loglikelihood user model (H0) -31539.117

Loglikelihood unrestricted model (H1) NA

Akaike (AIC) 63260.233

Bayesian (BIC) 63711.539

Sample-size adjusted Bayesian (SABIC) 63422.509

Root Mean Square Error of Approximation:

RMSEA 0.051

90 Percent confidence interval - lower 0.048

90 Percent confidence interval - upper 0.054

P-value H_0: RMSEA <= 0.050 0.330

P-value H_0: RMSEA >= 0.080 0.000

Robust RMSEA 0.054

90 Percent confidence interval - lower 0.050

90 Percent confidence interval - upper 0.057

P-value H_0: Robust RMSEA <= 0.050 0.035

P-value H_0: Robust RMSEA >= 0.080 0.000

Standardized Root Mean Square Residual:

SRMR 0.046

Parameter Estimates:

Standard errors Standard

Information Observed

Observed information based on Hessian

Latent Variables:

Estimate Std.Err z-value P(>|z|) Std.lv Std.all

RNSP =~

RNSP1.1 1.000 0.726 0.748

RNSP2.1 1.220 0.055 22.386 0.000 0.886 0.749

RNSP3.1 0.891 0.047 19.086 0.000 0.647 0.651

RNSP4.1 1.079 0.052 20.698 0.000 0.783 0.714

RNSP5.1 0.932 0.047 19.711 0.000 0.677 0.683

CM =~

CM1.1 1.000 0.735 0.702

CM2.1 0.820 0.051 16.032 0.000 0.602 0.576

CM3.1 0.834 0.052 16.077 0.000 0.613 0.587

CM4.1 0.868 0.055 15.699 0.000 0.638 0.554

CM5.1 0.649 0.044 14.869 0.000 0.477 0.544

CS =~

CS1.1 1.000 0.671 0.694

CS2.1 0.904 0.044 20.583 0.000 0.606 0.733

CS3.1 1.213 0.051 23.615 0.000 0.813 0.862

CS4.1 1.048 0.048 21.685 0.000 0.703 0.786

CS5.1 0.632 0.051 12.352 0.000 0.424 0.430

SO =~

SLA1.1 1.000 0.550 0.576

SLA2.1 1.235 0.071 17.417 0.000 0.679 0.755

SLA3.1 1.105 0.067 16.484 0.000 0.607 0.688

SLA4.1 1.258 0.071 17.819 0.000 0.692 0.788

SLA5.1 1.395 0.082 17.085 0.000 0.767 0.743

SLA6.1 1.358 0.081 16.863 0.000 0.746 0.722

ACT =~

ACT1.1 1.000 0.590 0.726

ACT2.1 1.144 0.058 19.774 0.000 0.676 0.739

ACT3.1 0.309 0.058 5.366 0.000 0.182 0.194

ACT4.1 1.040 0.053 19.536 0.000 0.614 0.732

ACT5.1 0.692 0.057 12.104 0.000 0.408 0.437

ACT6.1 0.397 0.061 6.506 0.000 0.234 0.238

Covariances:

Estimate Std.Err z-value P(>|z|) Std.lv Std.all

RNSP ~~

CM 0.265 0.026 10.348 0.000 0.496 0.496

CS 0.161 0.020 7.942 0.000 0.332 0.332

SO 0.160 0.018 8.665 0.000 0.400 0.400

ACT 0.139 0.019 7.414 0.000 0.323 0.323

CM ~~

CS 0.361 0.028 12.851 0.000 0.734 0.734

SO 0.332 0.027 12.457 0.000 0.823 0.823

ACT 0.308 0.025 12.469 0.000 0.709 0.709

CS ~~

SO 0.289 0.024 12.282 0.000 0.784 0.784

ACT 0.290 0.022 12.981 0.000 0.732 0.732

SO ~~

ACT 0.216 0.019 11.473 0.000 0.667 0.667

Intercepts:

Estimate Std.Err z-value P(>|z|) Std.lv Std.all

.RNSP1.1 4.371 0.030 144.580 0.000 4.371 4.501

.RNSP2.1 4.035 0.037 109.391 0.000 4.035 3.410

.RNSP3.1 4.380 0.031 141.234 0.000 4.380 4.405

.RNSP4.1 4.243 0.034 124.088 0.000 4.243 3.870

.RNSP5.1 4.473 0.031 144.562 0.000 4.473 4.512

.CM1.1 3.589 0.033 108.917 0.000 3.589 3.427

.CM2.1 3.232 0.033 97.871 0.000 3.232 3.092

.CM3.1 3.717 0.033 112.508 0.000 3.717 3.559

.CM4.1 3.291 0.036 90.461 0.000 3.291 2.859

.CM5.1 4.232 0.028 152.682 0.000 4.232 4.829

.CS1.1 4.061 0.031 130.949 0.000 4.061 4.205

.CS2.1 3.620 0.027 136.068 0.000 3.620 4.374

.CS3.1 3.854 0.030 127.487 0.000 3.854 4.085

.CS4.1 3.940 0.029 136.754 0.000 3.940 4.404

.CS5.1 3.923 0.032 122.439 0.000 3.923 3.974

.SLA1.1 4.111 0.030 135.368 0.000 4.111 4.306

.SLA2.1 4.185 0.029 146.086 0.000 4.185 4.654

.SLA3.1 4.245 0.028 150.985 0.000 4.245 4.811

.SLA4.1 4.280 0.028 152.835 0.000 4.280 4.879

.SLA5.1 3.725 0.033 113.088 0.000 3.725 3.612

.SLA6.1 3.553 0.033 107.487 0.000 3.553 3.439

.ACT1.1 3.872 0.026 147.955 0.000 3.872 4.763

.ACT2.1 3.744 0.030 126.902 0.000 3.744 4.093

.ACT3.1 3.440 0.031 112.534 0.000 3.440 3.666

.ACT4.1 3.743 0.027 137.678 0.000 3.743 4.460

.ACT5.1 3.422 0.030 112.709 0.000 3.422 3.665

.ACT6.1 2.536 0.032 78.285 0.000 2.536 2.577

Variances:

Estimate Std.Err z-value P(>|z|) Std.lv Std.all

.RNSP1.1 0.416 0.024 17.055 0.000 0.416 0.441

.RNSP2.1 0.616 0.036 16.951 0.000 0.616 0.440

.RNSP3.1 0.570 0.030 19.308 0.000 0.570 0.577

.RNSP4.1 0.589 0.033 18.066 0.000 0.589 0.490

.RNSP5.1 0.524 0.028 18.645 0.000 0.524 0.533

.CM1.1 0.557 0.032 17.470 0.000 0.557 0.508

.CM2.1 0.730 0.037 19.901 0.000 0.730 0.668

.CM3.1 0.715 0.036 19.730 0.000 0.715 0.656

.CM4.1 0.918 0.046 20.168 0.000 0.918 0.693

.CM5.1 0.541 0.027 20.226 0.000 0.541 0.704

.CS1.1 0.483 0.025 19.275 0.000 0.483 0.518

.CS2.1 0.317 0.017 18.701 0.000 0.317 0.463

.CS3.1 0.229 0.016 14.026 0.000 0.229 0.257

.CS4.1 0.306 0.018 17.313 0.000 0.306 0.382

.CS5.1 0.794 0.038 21.113 0.000 0.794 0.815

.SLA1.1 0.609 0.029 20.753 0.000 0.609 0.669

.SLA2.1 0.348 0.019 18.427 0.000 0.348 0.431

.SLA3.1 0.410 0.021 19.630 0.000 0.410 0.527

.SLA4.1 0.291 0.017 17.469 0.000 0.291 0.378

.SLA5.1 0.476 0.026 18.503 0.000 0.476 0.447

.SLA6.1 0.510 0.027 18.738 0.000 0.510 0.478

.ACT1.1 0.312 0.019 16.616 0.000 0.312 0.473

.ACT2.1 0.380 0.023 16.221 0.000 0.380 0.454

.ACT3.1 0.847 0.039 21.508 0.000 0.847 0.962

.ACT4.1 0.327 0.020 16.380 0.000 0.327 0.464

.ACT5.1 0.705 0.034 20.598 0.000 0.705 0.809

.ACT6.1 0.914 0.043 21.181 0.000 0.914 0.943

RNSP 0.527 0.041 12.968 0.000 1.000 1.000

CM 0.540 0.047 11.576 0.000 1.000 1.000

CS 0.450 0.038 11.753 0.000 1.000 1.000

SO 0.302 0.032 9.336 0.000 1.000 1.000

ACT 0.349 0.029 11.879 0.000 1.000 1.000

***Social relationships t2***

lavaan 0.6.17 ended normally after 103 iterations

Estimator ML

Optimization method NLMINB

Number of model parameters 91

Used Total

Number of observations 1187 1346

Number of missing patterns 102

Model Test User Model:

Test statistic 1648.984

Degrees of freedom 314

P-value (Chi-square) 0.000

Model Test Baseline Model:

Test statistic 13512.795

Degrees of freedom 351

P-value 0.000

User Model versus Baseline Model:

Comparative Fit Index (CFI) 0.899

Tucker-Lewis Index (TLI) 0.887

Robust Comparative Fit Index (CFI) 0.897

Robust Tucker-Lewis Index (TLI) 0.885

Loglikelihood and Information Criteria:

Loglikelihood user model (H0) -36426.271

Loglikelihood unrestricted model (H1) NA

Akaike (AIC) 73034.542

Bayesian (BIC) 73496.748

Sample-size adjusted Bayesian (SABIC) 73207.698

Root Mean Square Error of Approximation:

RMSEA 0.060

90 Percent confidence interval - lower 0.057

90 Percent confidence interval - upper 0.063

P-value H_0: RMSEA <= 0.050 0.000

P-value H_0: RMSEA >= 0.080 0.000

Robust RMSEA 0.062

90 Percent confidence interval - lower 0.059

90 Percent confidence interval - upper 0.065

P-value H_0: Robust RMSEA <= 0.050 0.000

P-value H_0: Robust RMSEA >= 0.080 0.000

Standardized Root Mean Square Residual:

SRMR 0.050

Parameter Estimates:

Standard errors Standard

Information Observed

Observed information based on Hessian

Latent Variables:

Estimate Std.Err z-value P(>|z|) Std.lv Std.all

RNSP =~

RNSP1.2 1.000 0.752 0.742

RNSP2.2 1.363 0.051 26.591 0.000 1.026 0.824

RNSP3.2 1.055 0.046 23.042 0.000 0.794 0.708

RNSP4.2 1.174 0.051 22.994 0.000 0.883 0.737

RNSP5.2 0.956 0.044 21.517 0.000 0.719 0.686

CM =~

CM1.2 1.000 0.701 0.697

CM2.2 0.845 0.050 16.957 0.000 0.592 0.577

CM3.2 0.836 0.050 16.624 0.000 0.586 0.593

CM4.2 0.900 0.051 17.524 0.000 0.631 0.579

CM5.2 0.657 0.044 14.896 0.000 0.460 0.523

CS =~

CS1.2 1.000 0.726 0.732

CS2.2 0.895 0.037 24.519 0.000 0.650 0.763

CS3.2 1.094 0.041 26.685 0.000 0.794 0.833

CS4.2 0.981 0.040 24.682 0.000 0.712 0.782

CS5.2 0.749 0.044 17.129 0.000 0.544 0.538

SO =~

SLA1.2 1.000 0.601 0.631

SLA2.2 1.280 0.059 21.562 0.000 0.768 0.791

SLA3.2 1.111 0.056 19.732 0.000 0.667 0.706

SLA4.2 1.273 0.057 22.182 0.000 0.765 0.833

SLA5.2 1.336 0.066 20.151 0.000 0.802 0.736

SLA6.2 1.177 0.061 19.193 0.000 0.707 0.685

ACT =~

ACT1.2 1.000 0.600 0.722

ACT2.2 1.174 0.054 21.724 0.000 0.705 0.749

ACT3.2 0.342 0.052 6.590 0.000 0.205 0.219

ACT4.2 1.065 0.050 21.473 0.000 0.639 0.754

ACT5.2 0.587 0.050 11.809 0.000 0.352 0.395

ACT6.2 0.549 0.058 9.493 0.000 0.330 0.323

Covariances:

Estimate Std.Err z-value P(>|z|) Std.lv Std.all

RNSP ~~

CM 0.180 0.022 8.281 0.000 0.342 0.342

CS 0.194 0.021 9.157 0.000 0.354 0.354

SO 0.167 0.018 9.085 0.000 0.370 0.370

ACT 0.099 0.017 5.709 0.000 0.219 0.219

CM ~~

CS 0.387 0.027 14.441 0.000 0.761 0.761

SO 0.331 0.024 13.760 0.000 0.787 0.787

ACT 0.292 0.022 13.063 0.000 0.693 0.693

CS ~~

SO 0.328 0.024 13.927 0.000 0.752 0.752

ACT 0.330 0.023 14.470 0.000 0.758 0.758

SO ~~

ACT 0.240 0.019 12.782 0.000 0.667 0.667

Intercepts:

Estimate Std.Err z-value P(>|z|) Std.lv Std.all

.RNSP1.2 4.289 0.030 144.817 0.000 4.289 4.228

.RNSP2.2 3.906 0.036 107.358 0.000 3.906 3.136

.RNSP3.2 4.253 0.033 129.770 0.000 4.253 3.793

.RNSP4.2 4.105 0.035 117.033 0.000 4.105 3.425

.RNSP5.2 4.375 0.031 142.426 0.000 4.375 4.172

.CM1.2 3.355 0.030 113.123 0.000 3.355 3.335

.CM2.2 3.137 0.030 103.345 0.000 3.137 3.058

.CM3.2 3.695 0.029 126.412 0.000 3.695 3.740

.CM4.2 3.111 0.032 96.498 0.000 3.111 2.853

.CM5.2 4.108 0.026 157.364 0.000 4.108 4.670

.CS1.2 3.880 0.030 131.039 0.000 3.880 3.912

.CS2.2 3.501 0.025 137.601 0.000 3.501 4.112

.CS3.2 3.666 0.028 128.872 0.000 3.666 3.849

.CS4.2 3.765 0.027 137.982 0.000 3.765 4.134

.CS5.2 3.784 0.030 124.165 0.000 3.784 3.747

.SLA1.2 3.970 0.028 140.496 0.000 3.970 4.174

.SLA2.2 4.029 0.029 139.662 0.000 4.029 4.148

.SLA3.2 4.232 0.028 150.375 0.000 4.232 4.480

.SLA4.2 4.133 0.027 150.900 0.000 4.133 4.502

.SLA5.2 3.567 0.033 109.636 0.000 3.567 3.273

.SLA6.2 3.432 0.031 111.432 0.000 3.432 3.328

.ACT1.2 3.734 0.025 149.667 0.000 3.734 4.494

.ACT2.2 3.645 0.028 128.876 0.000 3.645 3.875

.ACT3.2 3.503 0.029 122.792 0.000 3.503 3.730

.ACT4.2 3.582 0.026 140.200 0.000 3.582 4.225

.ACT5.2 3.314 0.027 122.698 0.000 3.314 3.720

.ACT6.2 2.583 0.031 82.777 0.000 2.583 2.528

Variances:

Estimate Std.Err z-value P(>|z|) Std.lv Std.all

.RNSP1.2 0.463 0.024 19.322 0.000 0.463 0.450

.RNSP2.2 0.498 0.031 15.960 0.000 0.498 0.321

.RNSP3.2 0.627 0.031 20.042 0.000 0.627 0.499

.RNSP4.2 0.657 0.034 19.378 0.000 0.657 0.457

.RNSP5.2 0.582 0.028 20.458 0.000 0.582 0.529

.CM1.2 0.520 0.029 18.082 0.000 0.520 0.514

.CM2.2 0.702 0.033 21.079 0.000 0.702 0.667

.CM3.2 0.633 0.030 20.887 0.000 0.633 0.648

.CM4.2 0.791 0.038 20.722 0.000 0.791 0.665

.CM5.2 0.562 0.026 21.656 0.000 0.562 0.726

.CS1.2 0.457 0.023 20.227 0.000 0.457 0.464

.CS2.2 0.303 0.016 19.525 0.000 0.303 0.418

.CS3.2 0.277 0.016 16.963 0.000 0.277 0.305

.CS4.2 0.323 0.017 18.909 0.000 0.323 0.389

.CS5.2 0.724 0.033 22.168 0.000 0.724 0.710

.SLA1.2 0.544 0.025 21.870 0.000 0.544 0.601

.SLA2.2 0.353 0.018 19.177 0.000 0.353 0.374

.SLA3.2 0.447 0.021 20.890 0.000 0.447 0.501

.SLA4.2 0.258 0.015 17.264 0.000 0.258 0.306

.SLA5.2 0.544 0.027 20.271 0.000 0.544 0.458

.SLA6.2 0.564 0.027 20.929 0.000 0.564 0.530

.ACT1.2 0.330 0.018 18.191 0.000 0.330 0.478

.ACT2.2 0.388 0.023 17.238 0.000 0.388 0.438

.ACT3.2 0.840 0.036 23.025 0.000 0.840 0.952

.ACT4.2 0.310 0.018 17.075 0.000 0.310 0.431

.ACT5.2 0.670 0.030 22.424 0.000 0.670 0.844

.ACT6.2 0.935 0.041 22.564 0.000 0.935 0.896

RNSP 0.566 0.041 13.906 0.000 1.000 1.000

CM 0.491 0.041 12.015 0.000 1.000 1.000

CS 0.527 0.039 13.649 0.000 1.000 1.000

SO 0.361 0.032 11.314 0.000 1.000 1.000

ACT 0.360 0.028 12.773 0.000 1.000 1.000

***Social relationships t3***

lavaan 0.6.17 ended normally after 96 iterations

Estimator ML

Optimization method NLMINB

Number of model parameters 91

Used Total

Number of observations 1041 1346

Number of missing patterns 78

Model Test User Model:

Test statistic 1803.801

Degrees of freedom 314

P-value (Chi-square) 0.000

Model Test Baseline Model:

Test statistic 13312.620

Degrees of freedom 351

P-value 0.000

User Model versus Baseline Model:

Comparative Fit Index (CFI) 0.885

Tucker-Lewis Index (TLI) 0.872

Robust Comparative Fit Index (CFI) 0.884

Robust Tucker-Lewis Index (TLI) 0.870

Loglikelihood and Information Criteria:

Loglikelihood user model (H0) -33734.601

Loglikelihood unrestricted model (H1) NA

Akaike (AIC) 67651.201

Bayesian (BIC) 68101.464

Sample-size adjusted Bayesian (SABIC) 67812.435

Root Mean Square Error of Approximation:

RMSEA 0.068

90 Percent confidence interval - lower 0.065

90 Percent confidence interval - upper 0.071

P-value H_0: RMSEA <= 0.050 0.000

P-value H_0: RMSEA >= 0.080 0.000

Robust RMSEA 0.069

90 Percent confidence interval - lower 0.066

90 Percent confidence interval - upper 0.072

P-value H_0: Robust RMSEA <= 0.050 0.000

P-value H_0: Robust RMSEA >= 0.080 0.000

Standardized Root Mean Square Residual:

SRMR 0.055

Parameter Estimates:

Standard errors Standard

Information Observed

Observed information based on Hessian

Latent Variables:

Estimate Std.Err z-value P(>|z|) Std.lv Std.all

RNSP =~

RNSP1.3 1.000 0.863 0.784

RNSP2.3 1.158 0.045 25.476 0.000 0.999 0.789

RNSP3.3 0.971 0.042 23.347 0.000 0.838 0.725

RNSP4.3 1.051 0.046 22.635 0.000 0.907 0.750

RNSP5.3 0.917 0.043 21.115 0.000 0.791 0.687

CM =~

CM1.3 1.000 0.789 0.763

CM2.3 0.890 0.046 19.413 0.000 0.703 0.660

CM3.3 0.826 0.046 17.892 0.000 0.652 0.642

CM4.3 0.969 0.048 20.178 0.000 0.765 0.646

CM5.3 0.584 0.045 12.901 0.000 0.461 0.468

CS =~

CS1.3 1.000 0.749 0.735

CS2.3 0.891 0.038 23.510 0.000 0.668 0.762

CS3.3 1.085 0.042 25.583 0.000 0.813 0.828

CS4.3 0.978 0.041 23.970 0.000 0.733 0.787

CS5.3 0.787 0.045 17.376 0.000 0.590 0.566

SO =~

SLA1.3 1.000 0.668 0.654

SLA2.3 1.262 0.059 21.502 0.000 0.843 0.795

SLA3.3 1.095 0.055 19.727 0.000 0.731 0.713

SLA4.3 1.222 0.057 21.483 0.000 0.816 0.800

SLA5.3 1.324 0.063 21.128 0.000 0.885 0.785

SLA6.3 1.191 0.059 20.181 0.000 0.796 0.737

ACT =~

ACT1.3 1.000 0.632 0.731

ACT2.3 1.094 0.052 20.979 0.000 0.691 0.735

ACT3.3 0.359 0.050 7.132 0.000 0.227 0.247

ACT4.3 1.025 0.048 21.553 0.000 0.647 0.775

ACT5.3 0.672 0.050 13.466 0.000 0.424 0.468

ACT6.3 0.550 0.054 10.108 0.000 0.347 0.355

Covariances:

Estimate Std.Err z-value P(>|z|) Std.lv Std.all

RNSP ~~

CM 0.273 0.029 9.288 0.000 0.401 0.401

CS 0.267 0.027 9.896 0.000 0.414 0.414

SO 0.231 0.025 9.374 0.000 0.400 0.400

ACT 0.155 0.022 7.008 0.000 0.285 0.285

CM ~~

CS 0.433 0.031 14.093 0.000 0.732 0.732

SO 0.388 0.029 13.486 0.000 0.736 0.736

ACT 0.280 0.024 11.548 0.000 0.561 0.561

CS ~~

SO 0.396 0.029 13.818 0.000 0.791 0.791

ACT 0.348 0.025 13.749 0.000 0.735 0.735

SO ~~

ACT 0.264 0.022 12.089 0.000 0.626 0.626

Intercepts:

Estimate Std.Err z-value P(>|z|) Std.lv Std.all

.RNSP1.3 4.139 0.034 120.086 0.000 4.139 3.760

.RNSP2.3 3.808 0.040 95.981 0.000 3.808 3.008

.RNSP3.3 4.186 0.036 115.565 0.000 4.186 3.623

.RNSP4.3 4.028 0.038 106.166 0.000 4.028 3.333

.RNSP5.3 4.259 0.036 117.736 0.000 4.259 3.696

.CM1.3 3.177 0.032 98.802 0.000 3.177 3.070

.CM2.3 2.936 0.033 88.482 0.000 2.936 2.759

.CM3.3 3.524 0.032 111.256 0.000 3.524 3.469

.CM4.3 2.864 0.037 77.665 0.000 2.864 2.420

.CM5.3 4.008 0.031 130.158 0.000 4.008 4.067

.CS1.3 3.780 0.032 118.485 0.000 3.780 3.706

.CS2.3 3.398 0.027 123.790 0.000 3.398 3.876

.CS3.3 3.514 0.031 114.387 0.000 3.514 3.579

.CS4.3 3.649 0.029 125.049 0.000 3.649 3.919

.CS5.3 3.681 0.033 112.408 0.000 3.681 3.531

.SLA1.3 3.753 0.032 117.732 0.000 3.753 3.673

.SLA2.3 3.818 0.033 115.364 0.000 3.818 3.598

.SLA3.3 4.066 0.032 126.999 0.000 4.066 3.964

.SLA4.3 3.927 0.032 122.889 0.000 3.927 3.846

.SLA5.3 3.356 0.035 95.197 0.000 3.356 2.979

.SLA6.3 3.222 0.034 95.530 0.000 3.222 2.986

.ACT1.3 3.606 0.027 133.275 0.000 3.606 4.175

.ACT2.3 3.584 0.029 121.639 0.000 3.584 3.815

.ACT3.3 3.470 0.029 120.052 0.000 3.470 3.780

.ACT4.3 3.493 0.026 133.203 0.000 3.493 4.181

.ACT5.3 3.283 0.029 115.087 0.000 3.283 3.624

.ACT6.3 2.541 0.031 81.794 0.000 2.541 2.595

Variances:

Estimate Std.Err z-value P(>|z|) Std.lv Std.all

.RNSP1.3 0.467 0.028 16.544 0.000 0.467 0.385

.RNSP2.3 0.604 0.037 16.491 0.000 0.604 0.377

.RNSP3.3 0.633 0.034 18.413 0.000 0.633 0.474

.RNSP4.3 0.639 0.037 17.483 0.000 0.639 0.437

.RNSP5.3 0.702 0.037 19.166 0.000 0.702 0.529

.CM1.3 0.448 0.029 15.261 0.000 0.448 0.418

.CM2.3 0.639 0.033 19.088 0.000 0.639 0.564

.CM3.3 0.607 0.032 19.086 0.000 0.607 0.588

.CM4.3 0.815 0.044 18.666 0.000 0.815 0.582

.CM5.3 0.759 0.036 20.999 0.000 0.759 0.781

.CS1.3 0.479 0.025 19.426 0.000 0.479 0.460

.CS2.3 0.323 0.017 18.933 0.000 0.323 0.420

.CS3.3 0.303 0.018 16.794 0.000 0.303 0.314

.CS4.3 0.330 0.018 18.127 0.000 0.330 0.380

.CS5.3 0.739 0.035 21.260 0.000 0.739 0.680

.SLA1.3 0.597 0.029 20.760 0.000 0.597 0.572

.SLA2.3 0.415 0.023 18.330 0.000 0.415 0.368

.SLA3.3 0.517 0.026 19.952 0.000 0.517 0.492

.SLA4.3 0.376 0.021 18.079 0.000 0.376 0.360

.SLA5.3 0.487 0.026 18.495 0.000 0.487 0.384

.SLA6.3 0.531 0.027 19.389 0.000 0.531 0.456

.ACT1.3 0.347 0.020 17.327 0.000 0.347 0.465

.ACT2.3 0.405 0.024 17.162 0.000 0.405 0.459

.ACT3.3 0.791 0.036 22.161 0.000 0.791 0.939

.ACT4.3 0.279 0.018 15.764 0.000 0.279 0.399

.ACT5.3 0.641 0.030 21.163 0.000 0.641 0.781

.ACT6.3 0.838 0.039 21.614 0.000 0.838 0.874

RNSP 0.745 0.053 13.986 0.000 1.000 1.000

CM 0.623 0.048 12.979 0.000 1.000 1.000

CS 0.562 0.043 13.101 0.000 1.000 1.000

SO 0.446 0.040 11.272 0.000 1.000 1.000

ACT 0.399 0.032 12.462 0.000 1.000 1.000

***Motivation (extrinsic and intrinsic) t1***

lavaan 0.6.17 ended normally after 37 iterations

Estimator ML

Optimization method NLMINB

Number of model parameters 25

Used Total

Number of observations 1124 1346

Number of missing patterns 24

Model Test User Model:

Test statistic 136.407

Degrees of freedom 19

P-value (Chi-square) 0.000

Model Test Baseline Model:

Test statistic 3076.261

Degrees of freedom 28

P-value 0.000

User Model versus Baseline Model:

Comparative Fit Index (CFI) 0.961

Tucker-Lewis Index (TLI) 0.943

Robust Comparative Fit Index (CFI) 0.961

Robust Tucker-Lewis Index (TLI) 0.943

Loglikelihood and Information Criteria:

Loglikelihood user model (H0) -12877.050

Loglikelihood unrestricted model (H1) NA

Akaike (AIC) 25804.100

Bayesian (BIC) 25929.716

Sample-size adjusted Bayesian (SABIC) 25850.309

Root Mean Square Error of Approximation:

RMSEA 0.074

90 Percent confidence interval - lower 0.063

90 Percent confidence interval - upper 0.086

P-value H_0: RMSEA <= 0.050 0.000

P-value H_0: RMSEA >= 0.080 0.217

Robust RMSEA 0.075

90 Percent confidence interval - lower 0.064

90 Percent confidence interval - upper 0.087

P-value H_0: Robust RMSEA <= 0.050 0.000

P-value H_0: Robust RMSEA >= 0.080 0.270

Standardized Root Mean Square Residual:

SRMR 0.062

Parameter Estimates:

Standard errors Standard

Information Observed

Observed information based on Hessian

Latent Variables:

Estimate Std.Err z-value P(>|z|) Std.lv Std.all

IM =~

INT1.1 1.000 0.818 0.750

INT2.1 1.314 0.048 27.376 0.000 1.076 0.935

INT3.1 1.156 0.044 26.560 0.000 0.946 0.797

EM =~

EEX1.1 1.000 0.631 0.505

EEX2.1 1.549 0.102 15.242 0.000 0.978 0.791

EEX3.1 1.580 0.104 15.183 0.000 0.998 0.791

EEX4.1 1.110 0.093 11.983 0.000 0.701 0.497

EEX5.1 1.012 0.086 11.780 0.000 0.639 0.493

Covariances:

Estimate Std.Err z-value P(>|z|) Std.lv Std.all

IM ~~

EM -0.089 0.020 -4.561 0.000 -0.173 -0.173

Intercepts:

Estimate Std.Err z-value P(>|z|) Std.lv Std.all

.INT1.1 3.363 0.033 102.016 0.000 3.363 3.080

.INT2.1 2.877 0.035 82.834 0.000 2.877 2.500

.INT3.1 2.818 0.036 78.537 0.000 2.818 2.375

.EEX1.1 2.595 0.037 69.354 0.000 2.595 2.076

.EEX2.1 2.357 0.037 63.515 0.000 2.357 1.905

.EEX3.1 2.231 0.038 58.973 0.000 2.231 1.768

.EEX4.1 2.953 0.042 69.608 0.000 2.953 2.096

.EEX5.1 2.925 0.039 75.091 0.000 2.925 2.257

Variances:

Estimate Std.Err z-value P(>|z|) Std.lv Std.all

.INT1.1 0.522 0.028 18.876 0.000 0.522 0.438

.INT2.1 0.167 0.029 5.756 0.000 0.167 0.126

.INT3.1 0.513 0.031 16.573 0.000 0.513 0.364

.EEX1.1 1.163 0.054 21.629 0.000 1.163 0.745

.EEX2.1 0.574 0.043 13.246 0.000 0.574 0.375

.EEX3.1 0.596 0.045 13.225 0.000 0.596 0.375

.EEX4.1 1.494 0.069 21.540 0.000 1.494 0.753

.EEX5.1 1.270 0.059 21.663 0.000 1.270 0.757

IM 0.670 0.049 13.782 0.000 1.000 1.000

EM 0.399 0.049 8.123 0.000 1.000 1.000

***Motivation (extrinsic and intrinsic) t2***

lavaan 0.6.17 ended normally after 44 iterations

Estimator ML

Optimization method NLMINB

Number of model parameters 25

Used Total

Number of observations 1214 1346

Number of missing patterns 20

Model Test User Model:

Test statistic 192.050

Degrees of freedom 19

P-value (Chi-square) 0.000

Model Test Baseline Model:

Test statistic 3741.190

Degrees of freedom 28

P-value 0.000

User Model versus Baseline Model:

Comparative Fit Index (CFI) 0.953

Tucker-Lewis Index (TLI) 0.931

Robust Comparative Fit Index (CFI) 0.953

Robust Tucker-Lewis Index (TLI) 0.931

Loglikelihood and Information Criteria:

Loglikelihood user model (H0) -13774.584

Loglikelihood unrestricted model (H1) NA

Akaike (AIC) 27599.168

Bayesian (BIC) 27726.710

Sample-size adjusted Bayesian (SABIC) 27647.300

Root Mean Square Error of Approximation:

RMSEA 0.087

90 Percent confidence interval - lower 0.076

90 Percent confidence interval - upper 0.098

P-value H_0: RMSEA <= 0.050 0.000

P-value H_0: RMSEA >= 0.080 0.845

Robust RMSEA 0.088

90 Percent confidence interval - lower 0.077

90 Percent confidence interval - upper 0.099

P-value H_0: Robust RMSEA <= 0.050 0.000

P-value H_0: Robust RMSEA >= 0.080 0.877

Standardized Root Mean Square Residual:

SRMR 0.071

Parameter Estimates:

Standard errors Standard

Information Observed

Observed information based on Hessian

Latent Variables:

Estimate Std.Err z-value P(>|z|) Std.lv Std.all

IM =~

INT1.2 1.000 0.876 0.785

INT2.2 1.254 0.038 33.316 0.000 1.099 0.953

INT3.2 1.099 0.036 30.496 0.000 0.963 0.805

EM =~

EEX1.2 1.000 0.640 0.523

EEX2.2 1.384 0.084 16.509 0.000 0.886 0.762

EEX3.2 1.671 0.098 17.079 0.000 1.069 0.848

EEX4.2 1.191 0.086 13.791 0.000 0.762 0.544

EEX5.2 0.699 0.072 9.728 0.000 0.448 0.347

Covariances:

Estimate Std.Err z-value P(>|z|) Std.lv Std.all

IM ~~

EM -0.175 0.022 -7.862 0.000 -0.311 -0.311

Intercepts:

Estimate Std.Err z-value P(>|z|) Std.lv Std.all

.INT1.2 3.139 0.032 97.413 0.000 3.139 2.811

.INT2.2 2.729 0.033 81.999 0.000 2.729 2.367

.INT3.2 2.645 0.035 76.479 0.000 2.645 2.212

.EEX1.2 2.552 0.035 72.595 0.000 2.552 2.086

.EEX2.2 2.151 0.033 64.261 0.000 2.151 1.850

.EEX3.2 2.201 0.036 60.635 0.000 2.201 1.746

.EEX4.2 2.937 0.040 72.597 0.000 2.937 2.095

.EEX5.2 2.728 0.037 73.274 0.000 2.728 2.114

Variances:

Estimate Std.Err z-value P(>|z|) Std.lv Std.all

.INT1.2 0.479 0.024 19.718 0.000 0.479 0.384

.INT2.2 0.122 0.023 5.307 0.000 0.122 0.092

.INT3.2 0.502 0.027 18.789 0.000 0.502 0.351

.EEX1.2 1.088 0.048 22.607 0.000 1.088 0.726

.EEX2.2 0.568 0.035 16.271 0.000 0.568 0.420

.EEX3.2 0.446 0.041 10.836 0.000 0.446 0.280

.EEX4.2 1.384 0.062 22.257 0.000 1.384 0.704

.EEX5.2 1.465 0.062 23.734 0.000 1.465 0.880

IM 0.768 0.049 15.623 0.000 1.000 1.000

EM 0.410 0.046 8.922 0.000 1.000 1.000

***Motivation (extrinsic and intrinsic) t3***

lavaan 0.6.17 ended normally after 38 iterations

Estimator ML

Optimization method NLMINB

Number of model parameters 25

Used Total

Number of observations 1041 1346

Number of missing patterns 19

Model Test User Model:

Test statistic 205.924

Degrees of freedom 19

P-value (Chi-square) 0.000

Model Test Baseline Model:

Test statistic 3374.943

Degrees of freedom 28

P-value 0.000

User Model versus Baseline Model:

Comparative Fit Index (CFI) 0.944

Tucker-Lewis Index (TLI) 0.918

Robust Comparative Fit Index (CFI) 0.944

Robust Tucker-Lewis Index (TLI) 0.918

Loglikelihood and Information Criteria:

Loglikelihood user model (H0) -11575.132

Loglikelihood unrestricted model (H1) NA

Akaike (AIC) 23200.265

Bayesian (BIC) 23323.963

Sample-size adjusted Bayesian (SABIC) 23244.560

Root Mean Square Error of Approximation:

RMSEA 0.097

90 Percent confidence interval - lower 0.085

90 Percent confidence interval - upper 0.109

P-value H_0: RMSEA <= 0.050 0.000

P-value H_0: RMSEA >= 0.080 0.992

Robust RMSEA 0.098

90 Percent confidence interval - lower 0.086

90 Percent confidence interval - upper 0.110

P-value H_0: Robust RMSEA <= 0.050 0.000

P-value H_0: Robust RMSEA >= 0.080 0.992

Standardized Root Mean Square Residual:

SRMR 0.078

Parameter Estimates:

Standard errors Standard

Information Observed

Observed information based on Hessian

Latent Variables:

Estimate Std.Err z-value P(>|z|) Std.lv Std.all

IM =~

INT1.3 1.000 0.895 0.807

INT2.3 1.199 0.035 34.270 0.000 1.073 0.956

INT3.3 1.052 0.034 31.362 0.000 0.942 0.839

EM =~

EEX1.3 1.000 0.696 0.569

EEX2.3 1.289 0.082 15.688 0.000 0.896 0.782

EEX3.3 1.328 0.083 15.945 0.000 0.924 0.754

EEX4.3 0.981 0.079 12.394 0.000 0.682 0.500

EEX5.3 0.720 0.072 10.070 0.000 0.501 0.408

Covariances:

Estimate Std.Err z-value P(>|z|) Std.lv Std.all

IM ~~

EM -0.176 0.026 -6.754 0.000 -0.283 -0.283

Intercepts:

Estimate Std.Err z-value P(>|z|) Std.lv Std.all

.INT1.3 2.887 0.034 83.695 0.000 2.887 2.601

.INT2.3 2.548 0.035 73.043 0.000 2.548 2.270

.INT3.3 2.431 0.035 69.559 0.000 2.431 2.165

.EEX1.3 2.626 0.038 69.038 0.000 2.626 2.147

.EEX2.3 2.186 0.036 61.357 0.000 2.186 1.908

.EEX3.3 2.205 0.038 57.925 0.000 2.205 1.800

.EEX4.3 2.986 0.042 70.291 0.000 2.986 2.187

.EEX5.3 2.566 0.038 67.262 0.000 2.566 2.089

Variances:

Estimate Std.Err z-value P(>|z|) Std.lv Std.all

.INT1.3 0.430 0.023 18.380 0.000 0.430 0.349

.INT2.3 0.108 0.020 5.366 0.000 0.108 0.086

.INT3.3 0.373 0.022 16.730 0.000 0.373 0.296

.EEX1.3 1.012 0.051 19.726 0.000 1.012 0.677

.EEX2.3 0.510 0.041 12.431 0.000 0.510 0.388

.EEX3.3 0.647 0.046 14.081 0.000 0.647 0.431

.EEX4.3 1.399 0.068 20.667 0.000 1.399 0.750

.EEX5.3 1.257 0.059 21.412 0.000 1.257 0.833

IM 0.802 0.053 15.255 0.000 1.000 1.000

EM 0.484 0.055 8.841 0.000 1.000 1.000

**Table C1.**

*List of Items (Translated from German into English)*

| **Code** | **Item** |
| --- | --- |
| *Student orientation* | |
| sSO1 | We students are friendly to each other. |
| sSO2 | The teacher is willing to give us tips (help us). |
| sSO3 | Making mistakes in class is allowed (okay). |
| sSO4 | The teacher is friendly to me. |
| sSO5 | The teacher gives enough praise during class. |
| sSO6 | I always have enough time to think during class. |
| *Clarity and structure* | |
| sCS1 | The teacher speaks in a way that I can understand all the words. |
| sCS2 | I always know what to do in class. |
| sCS3 | The teacher explains things in a way I can follow well. |
| sCS4 | I usually understand what we are working on. |
| sCS5 | In class, it is important to the teacher that everyone speaks clearly. |
| *Activation* | |
| sACT1 | I always work with concentration. |
| sACT2 | I contribute to the topic in class. |
| sACT3 | There are questions or tasks in class that really make me think. |
| sACT4 | I usually complete the tasks in class successfully. |
| sACT5 | I always have something to do in class (no waiting time). |
| sACT6 | I often present something in front of others during class. |
| *Classroom management* | |
| sCM1 | I can work in class without being disturbed. |
| sCM2 | The teacher always knows what is happening in the classroom. |
| sCM3 | The teacher has their materials ready and doesn’t have to search for them. |
| sCM4 | The noise level in class is such that I can work (learn) well. |
| sCM5 | I follow the rules in class. |
| *Absence of social problems with peers* | |
|  | In the past few weeks, did it happen that... |
| sNSP1 | ...you had problems with your class? * |
| sNSP2 | ...you had problems with individual classmates? * |
| sNSP3 | ...you felt like an outsider in your class? * |
| sNSP4 | ...you had problems with a school friend? * |
| sNSP5 | ...you couldn’t fall asleep because you were thinking about problems with a classmate? * |
