# Supplementary material for: Students' relationship quality in class: Exploring latent profiles, latent transitions and links to student motivation
Source: Br J Educ Psychol. 2025 Sep 10;95(4):1234–65. doi: 10.1111/bjep.70028 (PMC12590938; doi:10.1111/bjep.70028)
Supplement: Supplementary file 1 — Appendix S1.–S5. [file BJEP-95-1234-s001.zip › bjep70028-sup-0004-AppendixS4.docx]

**Appendix S4**

**Output D1**

*Missing Patterns*

EM

Deviance Residuals:

Min 1Q Median 3Q Max

-0.9454 -0.7635 -0.7406 1.4286 1.6898

Coefficients:

Estimate Std. Error z value Pr(>|z|)

(Intercept) -1.08349 0.13638 -7.945 0.00000000000000195 ***

Geschlecht_Dummy 0.50990 0.16075 3.172 0.00151 **

M_Status -0.06997 0.15338 -0.456 0.64823

---

IM

Deviance Residuals:

Min 1Q Median 3Q Max

-0.9663 -0.7658 -0.7450 1.4327 1.6837

Coefficients:

Estimate Std. Error z value Pr(>|z|)

(Intercept) -1.07667 0.13602 -7.915 0.00000000000000246 ***

Geschlecht_Dummy 0.55734 0.15957 3.493 0.000478 ***

M_Status -0.06324 0.15286 -0.414 0.679058

glm(formula = miss_SO3 ~ Geschlecht_Dummy + M_Status, family = "binomial",

data = datM)

SO

Deviance Residuals:

Min 1Q Median 3Q Max

-0.9713 -0.8082 -0.7454 1.4825 1.6831

Coefficients:

Estimate Std. Error z value Pr(>|z|)

(Intercept) -0.9512 0.1328 -7.164 0.000000000000784 ***

Geschlecht_Dummy 0.4450 0.1608 2.767 0.00566 **

M_Status -0.1874 0.1504 -1.246 0.21295

glm(formula = miss_CM3 ~ Geschlecht_Dummy + M_Status, family = "binomial",

data = datM)

CM

Deviance Residuals:

Min 1Q Median 3Q Max

-0.9511 -0.7914 -0.7437 1.4221 1.6855

Coefficients:

Estimate Std. Error z value Pr(>|z|)

(Intercept) -1.0004 0.1341 -7.458 0.000000000000088 ***

Geschlecht_Dummy 0.4415 0.1613 2.737 0.0062 **

M_Status -0.1436 0.1516 -0.947 0.3436

CS

Deviance Residuals:

Min 1Q Median 3Q Max

-0.9740 -0.8148 -0.7663 1.4594 1.6545

Coefficients:

Estimate Std. Error z value Pr(>|z|)

(Intercept) -0.9323 0.1322 -7.050 0.00000000000179 ***

Geschlecht_Dummy 0.4329 0.1596 2.713 0.00667 **

M_Status -0.1429 0.1495 -0.956 0.33909

ACT

Deviance Residuals:

Min 1Q Median 3Q Max

-0.9761 -0.8215 -0.7613 1.4723 1.6613

Coefficients:

Estimate Std. Error z value Pr(>|z|)

(Intercept) -0.9131 0.1318 -6.928 0.00000000000427 ***

Geschlecht_Dummy 0.4192 0.1600 2.620 0.00881 **

M_Status -0.1770 0.1493 -1.186 0.23561

NSP

Deviance Residuals:

Min 1Q Median 3Q Max

-1.0003 -0.8013 -0.7583 1.4225 1.6655

Coefficients:

Estimate Std. Error z value Pr(>|z|)

(Intercept) -0.9715 0.1330 -7.302 0.000000000000283 ***

Geschlecht_Dummy 0.5394 0.1585 3.404 0.000664 ***

M_Status -0.1279 0.1501 -0.852 0.394224
