# Supplementary material for: Students' relationship quality in class: Exploring latent profiles, latent transitions and links to student motivation
Source: Br J Educ Psychol. 2025 Sep 10;95(4):1234–65. doi: 10.1111/bjep.70028 (PMC12590938; doi:10.1111/bjep.70028)

**Appendix S5**

**Output E1**

*Alternative LPA Solution with Three Profiles*

**LPA t1**

Mclust EEI (diagonal, equal volume and shape) model with 3 components:

log-likelihood n df BIC ICL

-8513.851 1346 22 -17186.21 -17565.09

Clustering table:

1 2 3

538 562 246


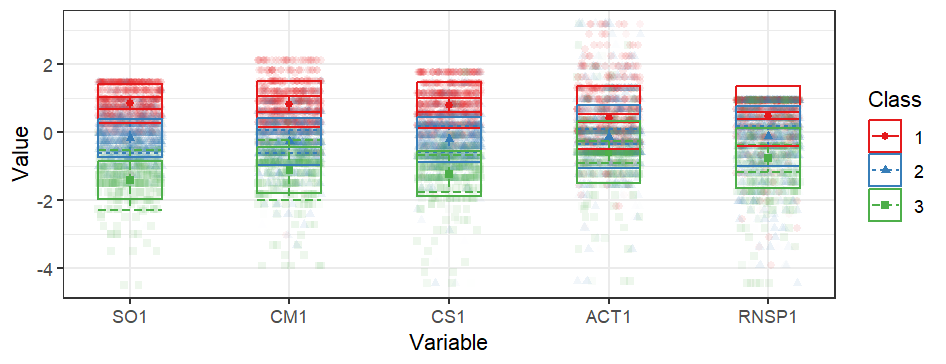


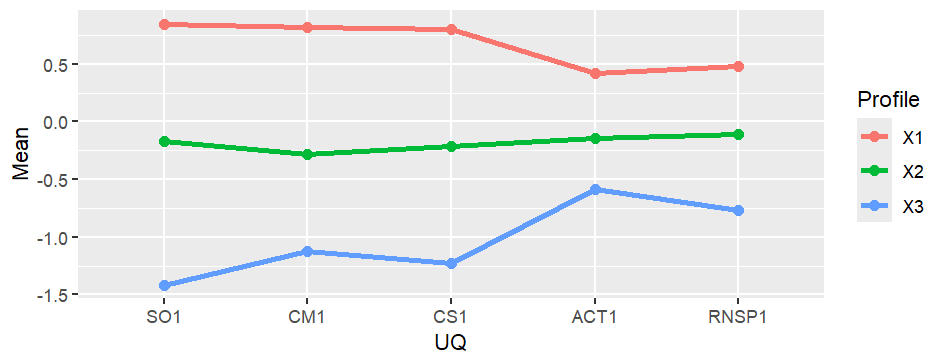


**LPA t2**

Mclust EEI (diagonal, equal volume and shape) model with 3 components:

log-likelihood n df BIC ICL

-8314.157 1346 22 -16786.82 -17056.42

Clustering table:

1 2 3

234 678 434


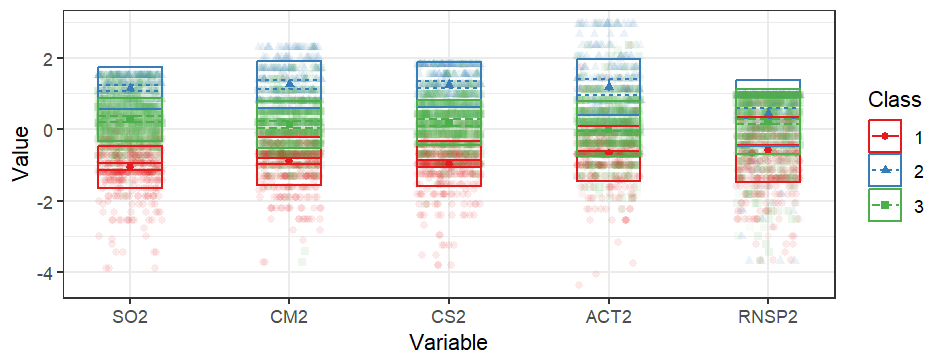


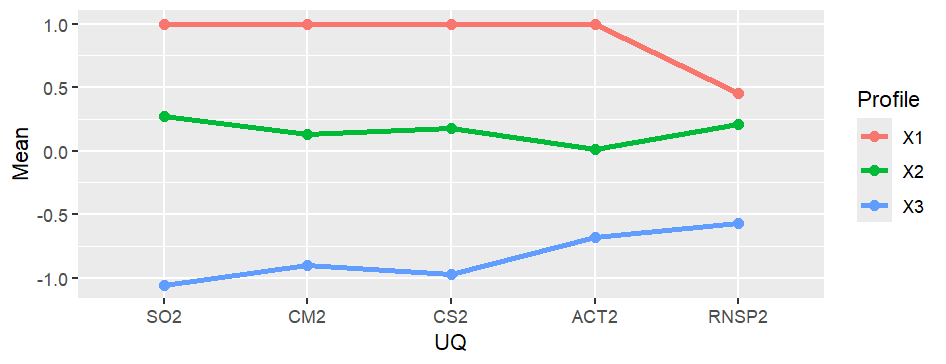


**LPA t3**

Mclust EEI (diagonal, equal volume and shape) model with 3 components:

log-likelihood n df BIC ICL

-8409.706 1346 22 -16977.92 -17185.94

Clustering table:

1 2 3

478 804 64


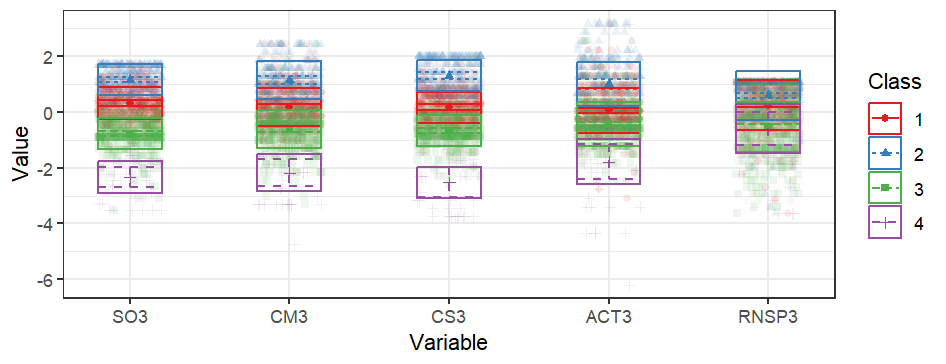


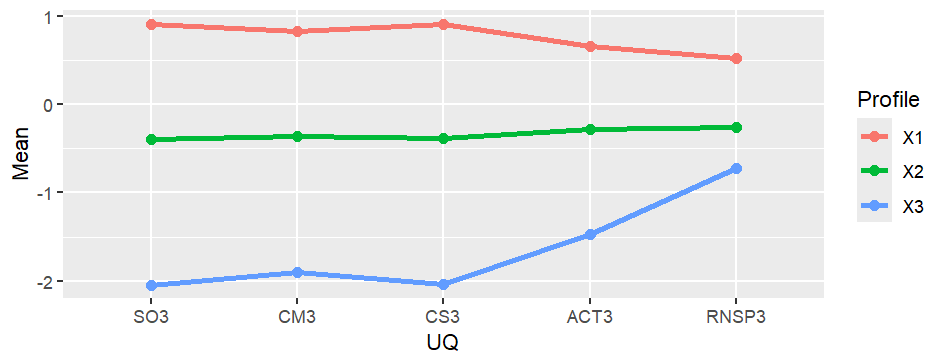

Supplement: Supplementary file 1 — Appendix S1.–S5. [file BJEP-95-1234-s001.zip › bjep70028-sup-0005-AppendixS5.docx]
